# Efficacy and safety of short-term use of a pelubiprofen CR and aceclofenac in patients with symptomatic knee osteoarthritis: A double-blinded, randomized, multicenter, active drug comparative, parallel-group, phase IV, non-inferiority clinical trial

**DOI:** 10.1371/journal.pone.0238024

**Published:** 2020-09-29

**Authors:** Joung Youp Shin, Moon Jong Chang, Myung Ku Kim, Seung-Baik Kang, Kang-Il Kim, Hee Gon Park, Sahnghoon Lee, Sung-Hwan Kim, Seung-Beom Han, Han Jun Lee, Young-Wan Moon, Jae-Doo Yoo

**Affiliations:** 1 Department of Orthopaedic Surgery, SMG-SNU Boramae Medical Center, Seoul National University College of Medicine, Seoul, Republic of Korea; 2 Department of Orthopaedic Surgery, Inha University Hospital, Incheon, Republic of Korea; 3 Department of Orthopaedic Surgery, School of Medicine, Kyung Hee University, Seoul, Republic of Korea; 4 Department of Orthopaedic Surgery, Dankook University Hospital, Cheonan-si, Chungcheongnam-do, Republic of Korea; 5 Department of Orthopaedic Surgery, Seoul National University College of Medicine, Seoul, Republic of Korea; 6 Department of Orthopaedic Surgery, Yonsei University College of Medicine, Gangnam Severance Hospital, Seoul, Republic of Korea; 7 Department of Orthopaedic Surgery, Korea University Medical Center, Seoul, Republic of Korea; 8 Department of Orthopaedic Surgery, Chung-Ang University, School of Medicine, Seoul, Republic of Korea; 9 Department of Orthopedic Surgery, Samsung Medical Center, Sungkyunkwan University School of Medicine, Seoul, Republic of Korea; 10 Department of Orthopaedic Surgery, Ewha Womans University School of Medicine, Ewha Medical Research Center, Seoul, Republic of Korea; Monash University, AUSTRALIA

## Abstract

**Introduction:**

At present, information about clinical efficacy and adverse events of controlled release (CR) form of pelubiprofen, a prodrug of 2-arylopropionic acid with relatively selective effects on cyclooxygenase-2 activity, remains scarce. In this study, we sought to determine non-inferiority of pelubiprofen CR 90 mg/day compared to aceclofenac 200 mg/day regarding clinical efficacy and adverse events after a 4-week course of medication in the patients with symptomatic knee osteoarthritis.

**Materials and methods:**

A total of 191 patients were randomly assigned to take either pelubiprofen CR 90 mg (n = 95) or aceclofenac 200 mg (n = 96). The primary outcome variable was non-inferiority of pain reduction between baseline and week 4 when assessed using a 100 mm pain visual analogue scale (VAS). Pelubiprofen was considered non-inferior to aceclofenac if the upper limit of the one-sided 97.5% confidence interval for the difference in terms of pain VAS was above 15 mm (the average change of pain VAS in the pelubiprofen group-pain VAS reduction in the aceclofenac group). Secondary outcome variables were the changes in 100 mm pain VAS at week 2 versus baseline, K-Western Ontario, and McMaster University Arthritis Index (K-WOMAC) changes at weeks 2 and 4 as compared to baseline, patient global assessment at weeks 2 and 4. The frequency and amount of rescue medicine usage at weeks 2 and 4 were also evaluated as the secondary outcome variable. For safety analysis, adverse events, clinical laboratory tests, vital signs, and physical examinations were assessed and conducted at each follow-up visit.

**Results:**

At week 4, the pain VAS values were significantly reduced in both groups receiving either pelubiprofen CR 90 mg or aceclofenac 200 mg as compared to the baseline. However, the pelubiprofen group and the aceclofenac group respectively showed the pain VAS changes of -22 and -21.9 in the pre-protocol set and -20.8 and -21.7 in the full analysis set, confirming non-inferiority. The pelubiprofen CR 90 mg showed a reduced incidence of adverse events compared to the aceclofenac 200 mg (p = 0.005).

**Conclusions:**

Pelubiprofen CR 90 mg is as effective as aceclofenac 200 mg with reduced adverse events for the treatment of symptomatic knee osteoarthritis.

## Introduction

Osteoarthritis (OA), one of the most common chronic diseases, causes symptoms such as pain and disability of joints in the elderly [1–3]. Patients with knee OA typically use medication to reduce the symptoms, although some patients with the end-stage OA require surgical treatment [4, 5]. Patients usually use non-steroidal anti-inflammatory drugs (NSAIDs) in the long-term, as NSAIDs do not alter the natural course of the knee OA [6, 7]. However, the long-term use of NSAIDs frequently causes adverse events (AEs) such as dyspepsia, abdominal pain, gastric ulcers, and renal damage [8]. Therefore, given that the patients with knee OA are typically elderly and thus have multiple comorbidities, both safety and efficacy of the NSAIDs are of essence.

Pelubiprofen, one of the 2-arylpropionic acid drugs, is structurally and pharmacologically related to ibuprofen [9]. Previously, it was reported that pelubiprofen has selective inhibitory effects on COX-2 activity [10]. In the study of the patients with rheumatoid arthritis (RA), pelubiprofen 90 mg showed non-inferiority to celecoxib 200 mg in terms of visual analogue scale (VAS) pain severity and AEs [9]. Furthermore, pelubiprofen 90 mg was not inferior to aceclofenac 200mg in terms of the degree of pain relief in the patients with chronic lower back pain [11]. Therefore, it can reasonably be predicted that pelubiprofen 90 mg would also have similar effect on the patients with knee OA without increase of AEs. However, previous research findings on the effect of controlled release (CR) from of pelubiprofen in the patients with knee OA remain scarce.

Among commonly used NSAIDs, aceclofenac is a phenylacetic acid derivative which is structurally related to diclofenac that showed a higher therapeutic index than other NSAIDs with similar analgesic activity [7, 12]. Furthermore, in a recent meta-analysis, aceclofenac was reported to have benefits over control analgesics in terms of function improvement. In this previous meta-analysis, aceclofenac showed benefits compared to diclofenac, and the difference between the two drugs corresponded to a difference in pain score of 1.15 cm on a 10-cm VAS scale [7]. In addition, aceclofenac showed reduced gastrointestinal (GI) AEs compared to other traditional NSAIDs [7].

Therefore, in the present study, we sought to evaluate non-inferiority of pelubiprofen CR 90 mg compared to aceclofenac 200 mg regarding clinical efficacy and AEs after a 4-week course of medication in the patients with symptomatic knee OA.

## Materials and methods

### Study participants

The patients visited from August 2015 to March 2016 and aged from 35 to 80 years old with symptomatic, primary knee OA were included in this prospective randomized controlled trial. Inclusion criteria were as follows: 1) diagnosis with knee OA according to the American College of Rheumatology (ACR) guidelines; 2) the Kellgren-Lawrens (K-L) grade I-III OA on knee radiographs; 3) stable knee pain for at least 3 months; 4) pain on a visual analogue scale (VAS) score of ≥ 40 mm after the NSAIDs/analgesic washout. This study included both femorotibial knee OA and patellofemoral knee OA patients. K-L grading was performed on the worse grading of the two compartments. Prospective participants were excluded if they had secondary OA, inflammatory arthritis such as RA, a history of surgery (including artificial knee joint surgery) and/or trauma in the knee joint within the previous 12 months, as well as had conditions that could confound the assessment of efficacy of the tested drug, such as tendinitis, bursitis, and fibromyalgia of the lower extremities. Furthermore, the patients with the following conditions were also excluded: a history of opioid use for over 3 months, intra-articular corticosteroid injection or systemic corticosteroid use (> 1500 μg) within the previous 3 months, intra-articular hyaluronic acid injection within previous 2 months, gastric ulcer or gastritis confirmed using endoscopy, genetic galactose intolerance, lactase deficiency or glucose-galactose uptake, contraindication for use of NSAID (such as allergic reactions), need of pain treatment regarding pre- or post-coronary artery bypass graft (CABG), active malignancy, ulcerative colitis, Crohn’s disease, active renal and/or liver diseases, significant hematologic, cardiac, pulmonary diseases, uncontrolled hypertension (systolic pressure ≥ 160 mmHg and/or diastolic pressure ≥ 100 mmHg), and substance dependence (alcohol or other drugs). Women of childbearing age who did not agree to use an effective method of birth control, had positive serum pregnancy results, and were lactating were also excluded. All participants signed written consent forms before participating in this clinical study.

### Sample size calculation

Power analysis of the designed trial showed that a sample size of 85 patients per group would be needed. To calculate sample size required to evaluate non-inferiority between pelubiprofen CR 90 mg and aceclofenac 200 mg for clinical efficacy, the following conditions were used: statistical power, 1-ß = 0.975; level of significance, a one-sided α = 0.025. We set the non-inferiority margin of the difference between pelubiprofen CR 90 mg and aceclofenac 200 mg to 15 mm of the VAS score ranging from 0 to 100 mm [13]. The sample size for each group required for a confidence interval of 95% and power of 97.5% was calculated using the following formula:

$$n_{t}=\frac{(\sigma_{t}^{2}+\sigma_{c}^{2}){\left(Z_{1-\alpha }+Z_{1-\beta }\right)}^{2}}{\delta^{2}}$$

In a previous study that compared aceclofenac and paracetamol in the management of symptomatic OA of the knee, the range of changes in 100 mm pain VAS score in the aceclofenac group was -18.34 ± 24.86 mm [14]. Therefore, for our sample size calculation, we set the standard deviation of 24.86. With these assumptions and estimating a dropout rate of 10%, the final sample size of 95 patients per group was obtained.

### Study design

The study was performed in the patients with knee osteoarthritis for 7 months as a multi-center, randomized, double-blinded, parallel-group, phase IV, and non-inferiority study. Researchers from 10 centers participated in this study from August 2015 to March 2016. All study protocols were approved by the institutional review boards of each hospital (DW_PlbCR_401) (SMG-SNU Boramae Medical Center, Inha University Hospital, Kyung Hee University Hospital at Gangdong, Dankook University Hospital, Seoul National University Hospital, Gangnam Severance Hospital, Korea University Medical Center, Chung-Ang University Hospital, Samsung Medical Center, and Ewha University Medical Center). This trial was registered with clinicalTrial.gov (NCT02682524). The patients were randomly assigned to one of the two study groups (pelubiprofen CR 90 mg or aceclofenac 200 mg) at the ratio of 1:1. Before the clinical trial, the randomization table (a sequential application of random numbers generated by the randomization program of SAS 9.2v system (SAS Institute, Cary, NC) starting from subject number 1) was generated by a statistician. The patients in the pelubiprofen group received pelubiprofen 90 mg (one 45 mg tablet, twice a day) for 4 weeks with an aceclofenac placebo tablet in the morning and evening. The patients in the aceclofenac group received 200 mg of aceclofenac (one 100 mg tablet, twice a day) for 4 weeks with a pelubiprofen placebo tablet in the morning and evening. Acetaminophen was used as the rescue medicine, and the patients were allowed to take one Tylenol ER sustained release tablet (acetaminophen 650 mg) as needed (but up to 3 tablets per day).

Fourteen days prior to the randomized assignment, the patients who had previously received NSAIDs, osteoarthritis nutrition, physiotherapy, and oriental medicine remedies had a drug wash-out period, which involved discontinuation of the administration and application of those drugs and therapies. The investigators maintained the double blind and performed the examination; the release of the double blind was considered on a case-by-case basis only in the event of a serious medical emergency, and the double blind was released when the information on the administration group affected the treatment of the patients.

### Efficacy and safety assessments

After the random assignment, follow-up visits were conducted on the days of baseline (week 0), and at weeks 2 and 4 after the drug administration. At the screening visit, we checked vital signs/weight and performed physical examinations, laboratory tests (complete blood count, electrolytes, blood urea nitrogen (BUN), creatinine, uric acid, total bilirubin, albumin, prothrombin time (PT), activated partial thromboplastin time (aPTT), urine human chorionic gonadotropin (hCG), urine protein, urine glucose), electrocardiogram (ECG), conventional radiographs and measured 100 mm pain VAS. At the baseline visit, we checked vital signs, performed weight measurements and physical examinations, and measured 100 mm pain VAS and K-WOMAC [15]. During the visit at 2 weeks after the administration, we checked vital signs, pain VAS, K-WOMAC, and did global patient assessment. During the visit at 4 weeks after the administration, we repeated laboratory tests, ECG, vital signs/weight measurements, physical examinations, pain VAS, K-WOMAC, and patient global assessment. The efficacy and safety of the treatment drug was assessed at weeks 0, 2, and 4. The primary outcome variable was the change in 100 mm pain VAS at week 4 as compared to the baseline. Secondary efficacy variables were the change in 100 mm pain VAS between week 2 and baseline, K-WOMAC score change between baseline and weeks 2 or 4, patient global assessment at weeks 2 and 4. The frequency and amount of rescue medicine usage at weeks 0, 2 and 4 were also assessed as the secondary outcome variable. We checked whether the drug administration was properly performed and the amount of the rescue medicine consumption at every visit. Global patient assessment was performed using a 5-point Likert-type scale regarding effectiveness of the medicine used (where 5 = excellent, 1 = very poor). Patients who rated their condition as excellent and good were categorized as the efficacy group. In addition, for safety analysis, adverse events, clinical laboratory tests, vital signs, and physical examination results were assessed. The AEs were categorized into the serious AEs, the drug-related AEs, and the discontinuation because of AEs.

### Statistical analyses

The data obtained from the subjects of the clinical trial were analyzed in three forms: safety set, full analysis (FA) set, and per protocol (PP) set. The safety set included all data from the patients who received at least one drug for the clinical trial. The FA set included all patients for whom the data on primary efficacy evaluation parameters were obtained after the administration of the drugs for the clinical trial among the participants who received the drugs for the clinical trial at least once. The PP set included data from the patients who had completed the drug administration in accordance with the protocol among the participants included in the FA set.

All the statistical significance tests were carried out by the two-sided test at a significance level (α) of 5%, and non-inferiority was tested using confidence interval. In the analysis of the demographic data and baseline characteristics data, continuous data were analyzed by independent t–test, while categorical data were analyzed by Chi-square test or Fisher's exact test. We assumed that pelubiprofen met the non-inferiority criterion if the upper limit of the one-sided 97.5% confidence interval for the difference in terms of pain VAS was below 15 mm (average changes of pain VAS in the pelubiprofen group-pain VAS reduction in the aceclofenac group). Additionally, a sensitivity analysis was performed using ANCOVA with factors of treatment group, site as a covariate for evaluating the treatment by center interaction. The differences in the K-WOMAC scale changes between clinical visits were determined using paired t-test. The results of global patient assessment were analyzed using Chi-square test or Fisher's exact test. For the use frequency of the rescue medicine, Chi-square test or Fisher's exact test and independent t-test were used for analysis. For safety confirmation, Chi-square test or Fisher's exact test was used to analyze the difference of the rate of adverse events. Laboratory tests, vital signs, and physical examination results were analyzed using paired t-test or Wilcoxon signed rank test.

## Results

### Patient characteristics

**Fig 1:**
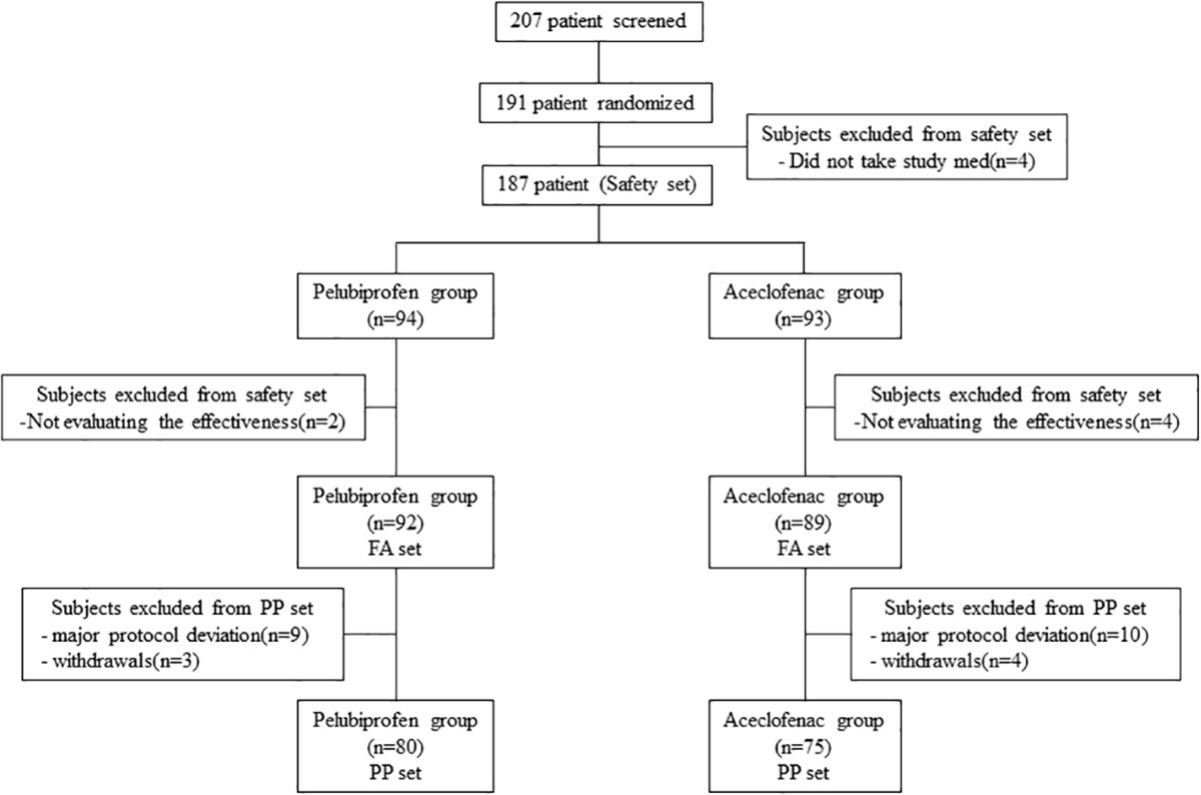
Flow chart of patient disposition.

A total of 191 patients who received the study medication at least once were randomized in the 10 centers (Fig 1). Among these, 4 patients withdrew their consent before taking the study drug. Therefore, we analyzed the safety set with the remaining 187 patients. The FA set consisted of all 181 patients for whom the data on primary efficacy variable were obtained before 6 patients (n = 2 in the pelubiprofen group and n = 4 in the aceclofenac group) withdrew their consent. The PP set (n = 155) excluded 19 patients with serious protocol violations (n = 9 in the pelubiprofen group and n = 10 in the aceclofenac group) or those who withdrew during the study period (n = 3 in the pelubiprofen group and n = 4 in the aceclofenac group). Serious protocol violations included inadequate inclusion/exclusion criteria (n = 6), taking the concomitant medication during the study period (n = 9), low adherence rate of taking study drugs below 70% (n = 4), and out of window period visits (n = 1), for duplicate counting.

The study population was predominantly female (73.3% {140 of 191 patients}) with the mean age of 62.1 years (standard deviation {SD}, 7.5 years; range, 43–77 years in the pelubiprofen group and 48–79 years in the aceclofenac group). Mean duration of knee OA was 3.2 years. The baseline characteristics were well balanced between the groups for the patients’ age, gender, laterality, K-L grade. The most common comorbidities were hypertension in both groups (Table 1).

**Table 1:** Demographic and baseline patient characteristics.

Parameters	Treatment groups	
	Pelubiprofen (n = 95)	Aceclofenac (n = 96)
Age (years)*	62.1 ± 7.8	62.1 ± 7.2
Sex, no. (%)		
Female	74 (78)	66 (69)
Male	21 (22)	30 (31)
Duration of OA (years)*	3.7 ± 4.0	2.7 ± 3.2
Laterality		
Right	52 (55)	53 (55)
Left	43 (45)	43 (45)
K-L grade		
Grade I	12 (13)	8 (8)
Grade II	40 (42)	53 (55)
Grade III	43 (45)	35 (37)
Comorbidities		
Hypertension	32 (34)	36 (38)
Chronic gastritis	1 (1)	2 (2)
Diabetes mellitus	12 (13)	13 (13)
Osteoporosis	5 (5)	5 (5)

Data are presented with number and percent in parenthesis.

* Data are presented with means and standard deviations. (mean ± standard deviation)

### Primary efficacy outcome

**Fig 2:**
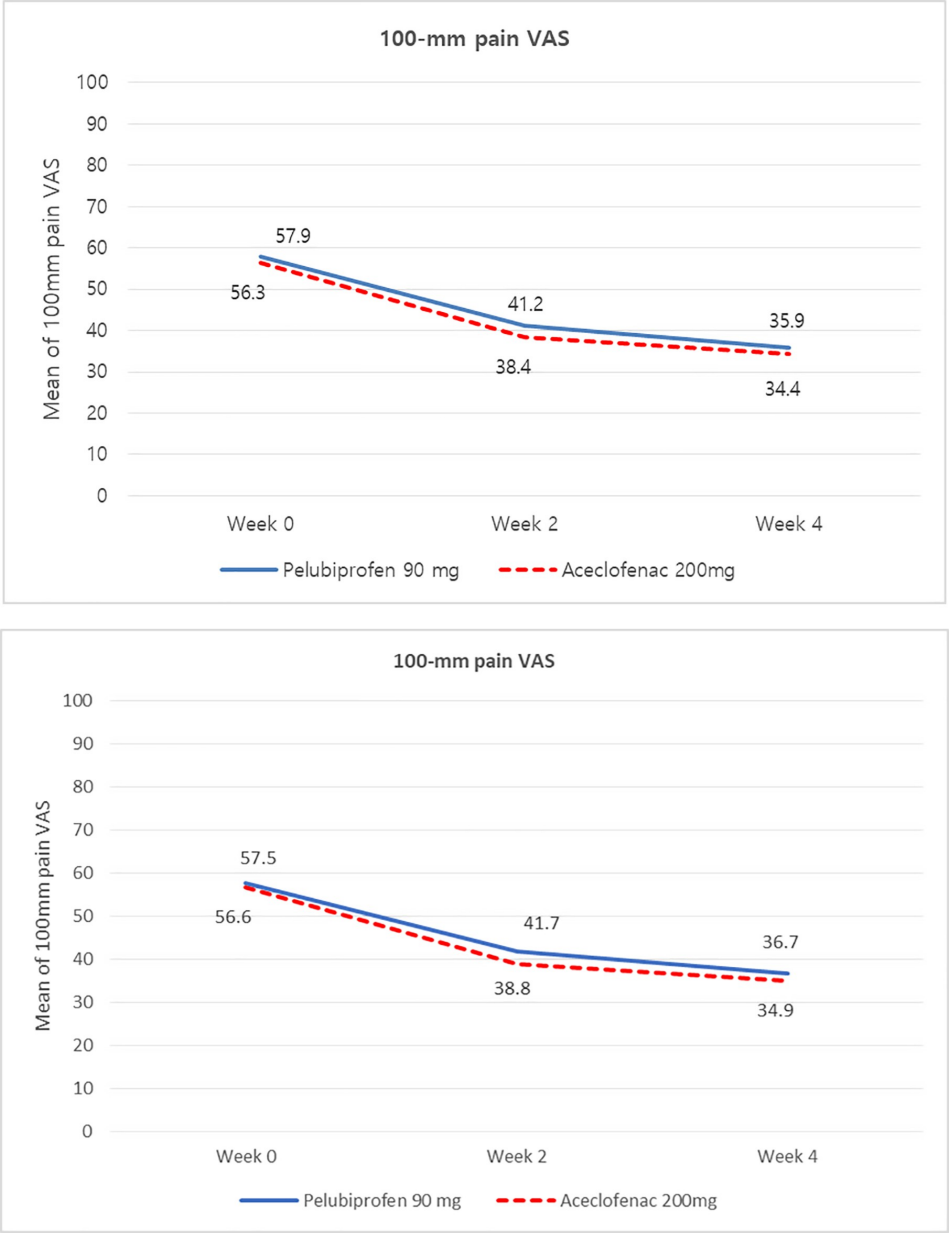
Efficacy outcome: Analysis of 100-mm pain VAS. A. Efficacy outcome: analysis of 100-mm pain VAS at week 2, 4 _ change of 100mm Pain VAS (PP). B. Efficacy outcome: analysis of 100-mm pain VAS at week 2, 4 _ change of 100mm Pain VAS (FA).

In the PP population, the 100 mm pain VAS score of the pelubiprofen group was 57.9 mm at week 0 and 35.9 mm at week 4. The mean change in pain VAS scores was −22.0 mm between weeks 0 and 4. In the aceclofenac group, 100 mm pain VAS score was 56.3 mm at week 0 and 34.4 mm at week 4. The mean change in pain VAS score was −21.9 mm between weeks 0 and 4 (Fig 2, Table 2). The difference of pain VAS changes between pelubiprofen group and aceclofenac group was -0.05 and the one sided 97.5% confidence interval (-4.56, 6.25) of the change difference met the criteria for non-inferiority. The FA population showed similar results to those of the PP population. (Table 2). The one-sided 97.5% confidence interval (-3.76, 6.64) of the change difference also met the criteria for non-inferiority. Additionally, there was no statistically significant interaction between the covariate and factor (treatment group, site.) (PP: p = 0.3625, FA: p = 0.3552) (Table 3).

**Table 2:** Primary efficacy outcome: Analysis of 100-mm pain VAS at week 4 (PP and FA sets).

Parameters	Treatment groups		Difference	
	Pelubiprofen 90 mg	Aceclofenac 200mg	Mean	One-sided 97.5% CI
PP set (Pelubiprofen [n = 80], Aceclofenac [n = 75])				
Week 0*	57.9 ± 11.9	56.3 ± 11.9		
Week 4*	35.9 ± 18.8	34.4 ± 16.5		
Differences (week 0 between week 4)*	-22.0 ± 18.1	-21.9 ± 18.3	-0.02	-4.56, 6.25
FA set (Pelubiprofen [n = 92], Aceclofenac [n = 89])				
Week 0*	57.5 ± 11.9	56.6 ± 11.8		
Week 4*	36.7 ± 19.0	34.9 ± 17.4		
Differences (week 0 between week 4)*	-20.8 ± 18.3	-21.7 ± 19.7	0.89	-3.76, 6.64

* Data are presented with means and standard deviations. (mean ± standard deviation) Abbreviations = PP, per protocol; FA, full analysis; CI, confidence interval

Adjusted by treatment group

Covariate: baseline mean of 100mm Pain VAS

**Table 3:** Primary efficacy outcome: analysis (adding site factor) of 100-mm pain VAS at week 4 (PP and FA sets).

Parameters	Treatment groups		Difference	
	Pelubiprofen 90 mg	Aceclofenac 200mg	Mean	one-sided 97.5% CI
PP set (Pelubiprofen [n = 80], Aceclofenac [n = 75])				
Week 0*	57.9 ± 11.9	56.3 ± 11.9		
Week 4*	35.9 ± 18.8	34.4 ± 16.5		
Differences (week 0 between week 4)*	-22.0 ± 18.1	-21.9 ± 18.3	-0.02	-4.72, 5.51
FA set (Pelubiprofen [n = 92], Aceclofenac [n = 89])				
Week 0*	57.5 ± 11.9	56.6 ± 11.8		
Week 4*	36.7 ± 19.0	34.9 ± 17.4		
Differences (week 0 between week 4)*	-20.8 ± 18.3	-21.7 ± 19.7	0.89	-3.89, 6.02

* Data are presented with means and standard deviations. (mean ± standard deviation) Abbreviations = PP, per protocol; FA, full analysis; CI, confidence interval

Adjusted by treatment group, site

Covariate: baseline mean of 100mm Pain VAS

### Secondary efficacy outcome

The change of the 100-mm pain VAS score from weeks 0 to 2 did not differ between the two groups (PP population, *p* = 0.410 and FA population, *p* = 0.253, respectively), and the one-sided 95% confidence interval (PP population, -2.76, 6.73 and FA population, -1.81, 6.81, respectively) of the change difference met the criteria for non-inferiority. In the change of K-WOMAC scores, no difference in any part and within any study period was observed in both PP and FA sets (Tables 4 and 5). In terms of patient global assessment and use of rescue medicine, there was no difference between the two groups in both PP and FA sets (Table 6).

**Table 4:** Analyses of secondary efficacy outcomes (PP set): 100-mm pain VAS at week 2 and K-WOMAC scores.

Parameters	Treatment groups		Difference	
	Pelubiprofen 90 mg	Aceclofenac 200mg	Mean	95% CI
100-mm pain VAS				
Week 0*	57.9±11.9	56.3±11.9		
Week 2*	41.2±15.7	38.4±16.3		
Differences (week 0 between week 2)*	-16.7±14.7	-17.9±17.2	1.22	-2.76, 6.73
K-WOMAC pain				
Week 0	8.1	7.8		
Week 2	6.0	5.0		
Week 4	5.9	4.7		
Differences (week 0 between week 2)*	-2.1±3.1	-2.8±2.6	0.7	-0.17, 1.64
Differences (week 0 between week 4)*	-2.2±3.7	-3.1±3.3	0.9	-0.22, 2.03
K-WOMAC stiffness				
Week 0	3.9	3.7		
Week 2	2.9	2.5		
Week 4	2.7	2.4		
Differences (week 0 between week 2)*	-1.0±1.5	-1.2±1.4	0.14	-0.33, 0.60
Differences (week 0 between week 4)*	-1.2±1.4	-1.3±1.6	0.07	-0.40, 0.54
K-WOMAC function				
Week 0	29.5	28.8		
Week 2	22.8	20.7		
Week 4	21.2	18.6		
Differences (week 0 between week 2)*	-6.7±11.2	-8.1±8.8	1.38	-1.80, 4.56
Differences (week 0 between week 4)*	-8.3±12.3	-10.2±10.5	1.87	-1.77, 5.52

* Data are presented with means and standard deviations. (mean ± standard deviation) The statistical analyses were performed using analysis of covariance (ANCOVA) test. Abbreviations: PP, per protocol; VAS, visual analogue scale; CI, confidence interval

**Table 5:** Analyses of secondary efficacy outcomes (FA set): 100-mm pain VAS at week 2 and K-WOMAC scores.

Parameters	Treatment groups		Difference	
	Pelubiprofen 90 mg	Aceclofenac 200mg	Mean	95% CI
100-mm pain VAS				
Week 0*	57.5±11.9	56.6±11.8		
Week 2*	41.7±15.6	38.8±15.5		
Differences (week 0 between week 2)*	-15.9±14.9	-17.9±16.8	1.95	-1.81, 6.81
K-WOMAC pain				
Week 0	8.0	7.7		
Week 2	6.1	5.1		
Week 4	6.0	4.8		
Differences (week 0 between week 2)*	-1.9±3.0	-2.7±2.5	0.75	-0.08, 1.57
Differences (week 0 between week 4)*	-2.0±3.6	-3.0±3.3	0.94	-0.08, 1.97
K-WOMAC stiffness				
Week 0	3.8	3.6		
Week 2	2.9	2.5		
Week 4	2.7	2.5		
Differences (week 0 between week 2)*	-1.0±1.6	-1.1±1.4	0.10	-0.35, 0.55
Differences (week 0 between week 4)*	-1.1±1.6	-1.1±1.6	0.04	-0.43, 0.50
K-WOMAC function				
Week 0	29.0	28.6		
Week 2	23.1	20.7		
Week 4	21.4	19.0		
Differences (week 0 between week 2)*	-6.1±11.6	-7.8±8.6	1.63	-1.37, 4.62
Differences (week 0 between week 4)*	-7.6±12.7	-9.7±10.8	2.10	-1.37, 5.56

* Data are presented with means and standard deviations. (mean ± standard deviation) The statistical analyses were performed using analysis of covariance (ANCOVA) test. Abbreviations: FA, full analysis; VAS, visual analogue scale; CI, confidence interval

**Table 6:** Secondary efficacy outcomes: patient global assessment and use of rescue medicine (PP and FA sets).

Parameters	Treatment groups		*p* value
	Pelubiprofen 90 mg	Aceclofenac 200mg	
PP set (Pelubiprofen [n = 80], Aceclofenac [n = 75])			
Patient global assessment			
Week 2			
Efficacy n (%)	61(76.3)	62(82.7)	0.626^†^
Non-efficacy n (%)	19(23.7)	13(17.3)	
Week 4			
Efficacy n (%)	57(71.3)	55(73.3)	0.772^†^
Non-efficacy n (%)	23(28.7)	20(26.7)	
Frequency^1^ of rescue medicine			
Week 0*	4.2±2.9	4.7±3.9	0.884*
Week 2*	4.1±3.0	4.2±3.0	0.914*
Week 4*	3.9±2.9	4.3±3.2	0.673*
Total dose^2^ of rescue medicine			
Week 0*	26.0±27.6	44.0±81.8	1.000*
Week 2*	25.6±30.8	25.8±33.0	0.849*
Week 4*	23.6±29.9	29.1±35.1	0.613*
FA set (Pelubiprofen [n = 92], Aceclofenac [n = 89])			
Patient global assessment			
Week 2			
Efficacy	66(72.5)	71(80.7)	0.198^†^
Non-efficacy	25(27.5)	17(19.3)	
Week 4			
Efficacy	62(67.4)	65(73.0)	0.407^†^
Non-efficacy	30(32.6)	24(27.0)	
Frequency^1^ of rescue medicine			
Week 0*	4.6±3.5	5.1±4.1	0.712*
Week 2*	4.6±3.2	4.1±2.9	0.583*
Week 4*	4.1±2.9	4.7±3.3	0.575*
Total dose^2^ of rescue medicine			
Week 0*	33.5±44.1	54.2±84.8	0.884*
Week 2*	31.0±35.5	24.9±31.2	0.623*
Week 4*	25.5±30.7	33.0±36.0	0.542*

* Data are presented with means and standard deviations. (mean ± standard deviation) ^1^Number of doses ^2^Total number of tablets

^†^Chi-square test, *Wilcoxon’s rank sum test. Abbreviations: PP, per protocol; FA, full analysis; CI, confidence interval

### Safety analysis

Forty-nine cases of AEs were reported from 35 patients (18.3%). Eleven AEs were reported from 10 patients (10.6%) in the pelubiprofen group, and 38 AEs were reported from 25 patients (26.9%) of the aceclofenac group. Among the 26 drug-related AEs, GI problems were the most frequent (14 cases in total; 7 cases of dyspepsia). The pelubiprofen group had a significantly reduced rate of drug-related AEs compared to the aceclofenac group (*p* = 0.011). Among the drug-related AEs, the pelubiprofen group showed a significantly lower rate of GI AEs than that in the aceclofenac group (3% vs 11%, *p* = 0.048) (Table 7).

**Table 7:** Adverse events during the study (safety set).

Parameters	Treatment groups				*p* value
	Pelubiprofen 90 mg (N = 94)		Aceclofenac 200mg (N = 93)		
	n(%)	events	n(%)	events	
Summary of all AEs					
Total AEs	10 (11)	11	25 (27)	38	0.005
serious AEs	1 (1)	1	0 (0)	0	1.000
drug-related AEs	5 (5)	6	16 (17)	20	0.011
discontinuation because of AEs	0 (0)	0	0 (0)	0	NA
Drug-related AEs					
Gastrointestinal disorder	3 (3)	3	10 (11)	10	0.048
Dyspepsia	0 (0)	0	6 (7)	6	
Upper abdominal pain	1 (1)	1	1 (1)	1	
Abdominal discomfort	1 (1)	1	0 (0)	0	
Dry mouth	1 (1)	1	0 (0)	0	
Epigastric discomfort	0 (0)	0	1 (1)	1	
Gastroesophageal reflux disease	0 (0)	0	1 (1)	1	
Nausea	0 (0)	0	1 (1)	1	
Urticaria	1 (1)	1	0 (0)	0	1.000
Face swelling	1 (1)	1	5 (5)	5	0.118
Increase of alanine aminotransferase	0 (0)	0	3 (3)	3	0.121

Data are presented with number and percent in the parenthesis. Abbreviations = AEs, adverse events, NA, not applicable

## Discussion

NSAIDs are the most commonly used medications for pain reduction and function improvement in the patients with symptomatic knee OA. Both safety and efficacy of NSAIDs are essential, as most patients with knee OA use the NSAIDs in the long term. In the present study, we sought to evaluate non-inferiority of short-term use of pelubiprofen CR to aceclofenac regarding clinical efficacy and rate of AEs in the highly selected sample of patients with symptomatic knee OA. The principal findings of this study were that non-inferiority of the pelubiprofen CR to aceclofenac was confirmed in terms of 100 mm pain VAS, K-WOMAC, and global patient assessment after a 4-week course of medication with a reduced rate of drug-related AEs.

The efficacy of traditional pelubiprofen 90 mg has been reported to be non-inferior to other control NSAIDs. Shin et al. compared traditional pelubiprofen 90 mg (30 mg three times daily) to aceclofenac 200 mg (100 mg twice daily) in the patients with lower back pain [11]. The authors reported that the mean changes in 100 mm pain VAS score of 4-week treatment were -30.0 ± 19.85 mm and -28.88 ± 17.86 mm in the pelubiprofen group and the aceclofenac group, respectively. Furthermore, in a 6-week, multicenter, randomized, double-blind study, Choi et al. compared the efficacy of the traditional pelubiprofen 90 mg with celecoxib 200 mg in the patients with RA. The results of this study showed that the pelubiprofen was non-inferior to celecoxib with a decrease in VAS pain severity (difference, 5.0 ± 20.1) [9]. The results of the present study are similar to previous reports in terms of clinical efficacy when comparing the CR form of pelubiprofen 90 mg and aceclofenac 200 mg in the patients with symptomatic knee OA. Therefore, our findings are largely consistent with previous research in this area.

However, despite confirming the reduced GI AEs rate of pelubiprofen CR as compared to aceclofenac in the present study, the rate of GI AEs in pelubiprofen remains a controversial issue. In a previous study in the patients with low back pain, the rate of heartburn and abdominal discomfort were similar between the pelubiprofen and aceclofenac groups [11]. However, in the study that compared the rate of AEs between pelubiprofen and celecoxib in RA patients, 50.6% reported 62 AEs in the pelubiprofen group, whereas 36.8% reported 33 AEs in the celecoxib group (*p* = 0.09). These findings suggest that further research is needed to confirm the GI safety of the pelubiprofen to treat OA. On the other hand, the CR form of a drug may reduce local gastrointestinal irritation caused by rapid localized dissolution of the traditional form of the drug. However, in a previous study that compared aceclofenac controlled release (CR) (200 mg once daily) to a traditional twice daily aceclofenac dose (100 mg twice daily), the patients with aceclofenac CR showed a higher rate of heartburn (10 of 45 vs. 3 of 45) even when there was no difference in terms of overall GI complications between the two groups [16]. The authors attributed the increased rate of heartburn in aceclofenac CR group to the fact that a single daily dose might cause a greater acidic insult to gastric mucosa [16]. In contrast, in the present study, the pelubiprofen CR group showed reduced GI AEs than the aceclofenac group. We were unable to fully explain these contradictory findings; however, given that there was no difference in the GI complication rate between traditional pelubiprofen and aceclofenac in a previous study [11], the CR form of pelubiprofen might have improved gastroprotective effects as compared to the traditional form of pelubiprofen. In a pelubiprofen CR tablet, crumbling is progressed from the surface of the drug, resulting in a slow release through the micropores. In addition, it was reported that pelubiprofen CR rarely dissolves in an environment where the pH is 1.2 [17]. Therefore, pelubiprofen CR tablets can hardly be diluted in the stomach for 2 hours, which could reduce gastric mucosal irritation. There were significant differences only in minor GI disorders, including dyspepsia among AE of pelubiprofen and aceclofenac in this study. In previous studies, traditional NSAIDs increases the risk of severe GI side effects by three to four times. [18] In the short term, even this minor problem may cause more severe side effects when taken for a long time.

The present study has several limitations. First, as we mentioned above, most patients with symptomatic knee OA use NSAIDs in the long term. However, in the present study, the drugs were prescribed only for 4 weeks. In general, NSAIDs are often taken for a long time in patients with arthritis, so a long period of study is needed. Therefore, in further research, it is necessary to evaluate the efficacy and safety of the pelubiprofen CR in the long-term period. Second, in our study, the patients with a higher risk of cardiovascular AEs were excluded. Therefore, we did not determine the safety of the pelubiprofen CR in the patients with increased cardiovascular risk. So studies on the cardiovascular risk of pelubiprofen and its use in patients with high cardiovascular risk are needed. Furthermore, since this study excluded patients with cardiovascular disease as well as many other serious diseases, there is a limitation that this study was conducted only for otherwise healthy patients. So patients with severe disease, as well as cardiovascular disease, included in the exclusion criteria of this study will need further long term studies, too. Third, the criterion for non-inferiority was rather large. However, the mean difference of 100 mm pain VAS between the pelubiprofen group and the aceclofenac group in the PP set analysis was only 0.02 (one sided 97.5% confidence interval, -4.56, 6.25). Therefore, the same outcome could have been achieved in terms of 100 mm pain VAS, although a stricter criterion was used.

In conclusion, the results of this clinical trial demonstrated that, in the patients with symptomatic knee OA, pelubiprofen CR 90 mg tablet was not inferior to aceclofenac 200 mg in terms of efficacy and number of adverse events.

## Supporting information

S1 Protocol (PDF)

S1 Checklist (DOC)

S1 Data (XLS)
