# Supplementary material for: Efficacy and safety of short-term use of a pelubiprofen CR and aceclofenac in patients with symptomatic knee osteoarthritis: A double-blinded, randomized, multicenter, active drug comparative, parallel-group, phase IV, non-inferiority clinical trial
Source: PLoS One. 2020 Sep 29;15(9):e0238024. doi: 10.1371/journal.pone.0238024 (PMC7523996; doi:10.1371/journal.pone.0238024)
Supplement: S1 Protocol — (PDF) [file pone.0238024.s001.pdf]

Clinical Trial Plan

A multicenter, randomized, double-blinded, parallel, active-controlled, phase IV clinical trial to evaluate the efficacy and safety of PlbCR and aceclofenac in the treatment of patients with osteoarthritis of the knee

**Clinical Trial Plan No. :**	DW_PlbCR_401
**Clinical Trial Plan Version/Date :**	Version 1.1_07 Oct 2015
**Study drug :**	Pelubi CR Tablet
**Clinical Trial Phase :**	Phase 4
**Clinical Trial Client :**	President of Daewon Pharmaceutical Co., Ltd. Seung-ho Baek<br>386, Cheonho-daero, Seongdong-gu, Seoul, Republic of Korea
**Clinical Trial Coordinator :**	Inha University Hospital Department of Orthopedics Professor Myeong-gu Kim
**Clinical Trial Period :**	12 Months from IRB approval date for clinical trial plan

Confidential

This document is the property of DAEWON PHARM and may not – in full or in part – be passed on, reproduced, published or otherwise used without the express permission of DAEWON PHARM

Table of Contents

▣ ACRONYMS AND TERMS 5

▣ OVERVIEW OF CLINICAL TRIAL PLAN 6

▣ CLINICAL TRIAL SCHEDULE 10

1. INTRODUCTION 12

1.1 Background 12

2. PURPOSE AND HYPOTHESIS 15

3. OVERALL DESIGN AND PLAN 16

3.1 Design 16

3.2 Number of Subjects and Basis of Calculation 17

3.2.1 Number of Subjects 17

3.2.2 Basis of Calculation 17

3.3 Participant Clinical Trial Institutions 18

3.4 Assignment of Administration Group 19

4. METHOD AND PROCEDURE OF ASSESSMENT 20

4.1 Method of Assessment 20

4.1.1 Effectiveness Assessment 20

4.1.2 Safety Assessment 21

4.1.3 Other 23

4.2 Procedure by Visit 24

4.2.1 Visit 1(Screening and Washout Period, 14 Days Prior to Trial) 24

4.2.2 Visit 2(Random Assignment and Administration of Investigational product, Day 1) 25

4.2.3 Visit 3(Intermediary Visit, Day 14±3) 25

4.2.4 Visit 4(End of Clinical Trial, Day 28±3) ... 26
4.2.5 Unscheduled Visit ... 26

5. SUBJECT SELECTION AND DROPOUT CRITERIA ... 27

5.1 Inclusion Criteria ... 27

5.2 Exclusion Criteria ... 27

5.3 Dropout and Disqualification Criteria ... 28

5.4 Clinical Trial Compliance and Action for Plan Violation ... 29

6. INVESTIGATIONAL PRODUCTS ... 31

6.1 Overview of Investigational products ... 31
6.1.1 Study drug ... 31
6.1.2 Control Drug ... 31
6.1.3 Study drug Placebo ... 31
6.1.4 Control Drug Placebo ... 31
6.1.5 Rescue drug ... 32

6.2 Dosage, Administration Method, Administration Period ... 32

6.3 Production, Packaging, and Labeling of Investigational products ... 32

6.4 Management of Investigational products ... 34

6.5 Maintenance and Cancellation of Double-blind ... 34

6.6 Compliance Assessment ... 35

6.7 Co-administered Drugs and Treatment ... 35
6.7.1 Permitted Drugs ... 35
6.7.2 Restricted Drugs ... 36

7.1 Evaluation Variables ... 38
7.1.1 Primary Effectiveness Evaluation Variables ... 38
7.1.2 Secondary Effectiveness Evaluation Variables ... 38
7.1.3 Safety Evaluation Variables ... 38

7.2 Plan for Statistical Analysis 38
7.2.1 Definition of Analysis Sets 38
7.2.2 General Statistical Principles 38
7.2.3 Demographic Data and Baseline Data Analysis 39
7.2.4 Effectiveness Analysis 39
7.2.5 Safety Analysis 41
7.2.6 Processing of Dropout or Missing Values 42
7.2.7 Scheduled Intermediary Analysis and Data Monitoring 42

8. ADVERSE EVENTS 43

8.1 Definition of Adverse Events 43

8.2 Collection and Record of Adverse Events 44

8.3 Assessment of Adverse Events 44
8.3.1 Assessment of Severity 45
8.3.2 Assessment of Cause and Effect Relationship 45

8.4 Report of Serious Adverse Events 46

8.5 Report of Suspected Unexpected Serious Adverse Reaction(SUSAR) and Countermeasure 47

8.6 Pregnancy 48

9. ETHICS AND ADMINISTRATIVE PROCEDURE 50

9.1 Clinical Trial Plan Compliance 50

9.2 Authorization and Revision of Clinical Trial Plan 50

9.3 Subject Consent Procedure 50

9.4 Plan for Subject Safety and Protection 52

9.5 Subject Consultation and Consultation Criteria after Clinical Trial 52

9.6 Regulation for Victim Compensation 52

9.7 Storage of Clinical Trial Related Documents and Records 52
9.7.1 Case Report and Evidentiary Materials... 52
9.7.2 Access of Evidentiary Materials 53
9.7.3 Storage of Clinical Trial Data.. 53
9.7.4 Inspection and On-site Survey 53

9.8 Confidentiality for Clinical Trial Documents and Subject Records 54

9.9 Monitoring of Clinical Trial Performing Institution 54

9.10 Stoppage of Clinical Trial.. 54

9.11 Clinical Trial Report and Publication 55

10. REFERENCES 56

11. APPENDIX 58

11.1 Expected Adverse Reactions and Use Precaution for Investigational product.. 58
11.1.1 Adverse Reactions and Use Precaution for Pelubi CR Tablet 58
11.1.2 Adverse Reactions and Use Precaution for Airtal Tablet 69
11.1.3 Adverse Reactions and Use Precaution for Tylenol ER Tablet......... 79

11.2 ACR Diagnosis Criteria... 84

11.4 K-WOMAC Scale.. 86

12. LIST OF ATTACHMENTS 88

▣ Acronyms and Terms

ACE	: Angiotensin Converting Enzyme
ADL	: Activities of Daily Living
ADR	: Adverse Drug Reaction
AE	: Adverse Event
ALT	: Alanine Aminotransferase
ALP	: Alkaline phosphatase
AST	: Aspartate Aminotransferase
β-hCG	: Beta unit of Human Chorionic gonadotropin
BUN	: Blood Urea Nitrogen
Cr	: Creatinine
CRA	: Clinical Research Associate
CRF	: Case Report Form
FAS	: Full Analysis Set
F/U	: Follow up
Hb	: Hemoglobin
Hct	: Hematocrit
IRB	: Institutional Review Board
LDH	: Lactate Dehydrogenase
LOCF	: Last Observation Carried Forward
NSAIDs	: Non-Steroidal Anti-Inflammatory Drug
OA	: Osteoarthritis
pH	: Negative logarithm of the hydrogen ion concentration
PPS	: Per Protocol Set
RBC	: Red Blood Cell
SGOT	: Serum Glutamic Oxaloacetic Transaminase
SGPT	: Serum Glutamic Pyruvic Transaminase
VAS	: Visual Analogue Scale
WBC	: White Blood Cell
WHO	: World Health Organization
WOMAC Scale	: Western Ontario MacMaster Questionnaire Scale
γ-GTP	: Gamma-Glutamyl Trans Peptidase

▣ Overview of Clinical Trial Plan

**Title**	A multicenter, randomized, double-blinded, parallel, active-controlled, phase IV clinical trial to evaluate the efficacy and safety of PlbCR and aceclofenac in the treatment of patients with osteoarthritis of the knee
**Client**	Daewon Pharmaceutical Co., Ltd. President Seung-ho Baek<br>386, Cheonho-daero, Seongdong-gu, Seoul, Republic of Korea
**Consigning Institution**	CliPS Co., Ltd. President Jun-hwan Ji<br>13F Police Mutual Aid Association Jaram Building, 78, Mapo-daero, Mapo-gu, Seoul, Republic of Korea
**Testing Institution and Supervisor**	Refer to Attachment 3. Clinical Trial Performing Institutions and Investagors
**Clinical Trial Period**	12 Months from IRB approval date
**Subject Disease**	Osteoarthritis of the knee
**Purpose**	The purpose of this clinical trial is to assess effect of pain relief and safety assessed by 100mm Pain VAS for each group after administering the Pelubi CR tablet and the aceclofenac tablet for 28 days to patients with osteoarthritis of the knee to prove that the effect of pain relief for Pelubi CR tablet is not inferior compared to that of aceclofenac tablet.
**Phases and Design**	This clinical trial is a multicenter, randomized, double-blinded, parallel, active-controlled, phase IV clinical trial.
**Method**	This clinical trial was designed as a multicenter, randomized, double-blinded, parallel, active-controlled, phase IV clinical trial.<br><br>Once the subject gives written consent to participate in the clinical trial, subjects who have been medicated with non-steroid anti-inflammatory pain reliever including narcotic analgesic, osteoarthritis supplements and treated with physical therapy and oriental therapy(Acupuncture, cupping, moxibustion) within 2 weeks prior to administration of clinical trial must undergo a washout period by stopping the administration and application of such treatments for 3~14 days prior to administration of investigational product. Screening period including the washout period cannot exceed 2 weeks.<br><br>Subjects who are not restricted in terms of prohibited medication and

▣ Overview of Clinical Trial Plan

<table>
<tr><td></td><td>therapy prior to screening visit can skip the washout period if they meet the subject qualification criteria and be administered with investigational product.
Subjects who are qualified as the result of qualification examination results according to the selection criteria after completing the washout period are randomly assigned at the ratio of 1:1 to control group or test group. Randomly assigned subjects are medicated with study drug or control drug for 28 days and they are evaluated in terms of effectiveness and safety by visiting the testing institution on day 14 and 28 after medication.</td></tr>
<tr><td>Investigational products</td><td>• Study drug: Pelubi CR tablet(Pelubiprofen 45mg)
• Control drug : Airtal tablet(Aceclofenac 100mg)
• Rescue drug : Tylenol ER tablet 650mg(Acetaminophen)</td></tr>
<tr><td>Dosage, Administration Route/Method/Period</td><td>• Study drug: Oral administration of 1 tablet after meal twice per day
• Control drug : Oral administration of 1 tablet after meal twice per day
<table>
<tr><th>Group</th><th>Morning</th><th>Evening</th></tr>
<tr><td>Test Group</td><td>1 Pelubi CR tablet + 1 Airtal tablet placebo</td><td>1 Pelubi CR tablet + 1 Airtal tablet placebo</td></tr>
<tr><td>Control Group</td><td>1 Pelubi CR tablet placebo + 1 Airtal tablet</td><td>1 Pelubi CR tablet placebo + 1 Airtal tablet</td></tr>
</table></td></tr>
<tr><td>Number of Subjects</td><td>(1)Level of significance, $\alpha=0.025$(One-sided test)
(2)Power of the test, $1-\beta=0.975$
(3)Test group to control group ratio, $\lambda=1$
(4)Standard deviation between test and control groups, $\sigma_t=\sigma_c=24.86$m
(5)Clinical tolerance for noninferiority test, $\delta=-15$mm
(6)Trace loss rate is assumed to be 10%(r=0.10)
<table>
<tr><th></th><th>Test Group</th><th>Control Group</th><th>Total Subjects</th></tr>
<tr><td>Number of cases for effectiveness assessment</td><td>85</td><td>85</td><td>170</td></tr>
<tr><td>Number of cases including 10% dropout rate</td><td>95</td><td>95</td><td>190</td></tr>
</table></td></tr>
</table>

▣ Overview of Clinical Trial Plan

**Inclusion Criteria**	1. Male and female patients between age 35~80<br>2. Patients diagnosed with osteoarthritis of the knee according to ACR diagnosis criteria with Kellgran & Lawrence stage Ⅰ~Ⅲ diagnosis on x-ray imaging<br>3. Patients evaluated with osteoarthritis to be stable for 3 months prior to clinical trial by clinical physician<br>4. Patient with at least 40mm on 100mm Pain VAS scale at visit 2<br>5. Patients who voluntarily gave consent in written form to participate in this clinical trial
**Exclusion Criteria**	1. Patient with secondary osteoarthritis<br>2. Patient suffering from disease that may affect the effectiveness assessment such as bursitis, tenosynovitis, fibromyalgia syndrome, rheumarthritis, and other inflammatory joint disease<br>3. Subject with confirmed with an ulcer or stomach disease through gastroscope<br>4. Patient with history of invasive knee joint operation, knee joint injury, arthroscopic joint operation within 12 months prior to screening visit or patient scheduled operation during clinical trial period<br>5. Patient with history of artificial joint surgery for knee joint<br>6. Patient with malignant tumor(However, patient without remission within 5 years after completion of treatment can be registered)<br>7. Patient with regular administration of psychoactive drug or narcotic analgesic which may affect sense of pain for at least 3 months<br>8. Subject administered with glenoid cavity injection or systemic injection(Oral administration or absorption of corticosteroid for 1 day exceeding 1500㎍) of steroid within 3 months prior to screening visit<br>9. Subject with hyaluronic acid injection for knee joint within 2 months prior to screening visit<br>10. Subject with intercurrent disease or medicated with co-administered drugs that are prohibits prescription of non-steroid anti-inflammatory pain reliever<br>11. Patient with history of asthma, hives, and allergic reaction against aspirin or other non-steroid anti-inflammatory pain reliever(COX-2 inhibitor included)

▣ Overview of Clinical Trial Plan

	12. Patient requiring treatment for pain before and after CABG<br>13. Patient with inherited galactose intolerance, lapp deficiency, or glucose-galactose malabsorption<br>14. Patient with inflammatory bowel disease such as Crohn's disease or ulcerative colitis<br>15. Patient with severe renal disorder<br>16. Patient with severe liver disorder<br>17. Severe uncontrollable high blood pressure(Systolic blood pressure≥160mmHg, diastolic blood pressure≥100mmHg)<br>18. Patient with clinically significant systemic disease such as hematologic, heart(Congestive heart failure, ischemic heart disease, etc.), lung, nervous disease<br>19. Patient who does not consent for birth control* method permitted for this clinical trial among female subjects that may become pregnant during clinical trial period<br>*Medically permitted birth control method: Condom, usage of intravenous and inserted contraceptive, installation of birth control device within the vagina, oral birth contraceptive<br>20. Subjects with dependency of drugs or alcohol<br>21. Other patients disqualified by the researcher
**Effectiveness Assessment**	• **Primary Effectiveness Evaluation Variable :**<br>1) Change in 100mm Pain VAS on 28th day compared to baseline<br>• **Secondary Effectiveness Evaluation Variables :**<br>1) Change in 100mm Pain VAS on 14th day compared to baseline<br>2) Change in K-WOMAC Scale on 14th and 28th days compared to baseline<br>3) Patient Global Assessment<br>4) Frequency of rescue drug usage
**Safety Assessment**	• Adverse events<br>• Vital signs<br>• Laboratory tests<br>• Physical examinations

▣ Clinical Trial Schedule

	Washout Period[1]	Administration Period		
Visit	Visit 1	Visit 2	Visit 3	Visit 4
Day	Within 14 days before	Day 1	Day 14	Day 28
Visit Window			±3 day	±3 day
Written consent form	X			
Demographic data	X			
Medical/Medication history	X	X		
Physical examination	X	X	X	X
Vital signs / Weight	X	X	X	X
ECG[3]	X			X
Laboratory Tests[4]	X[5]			X
X-ray examination[6]	X			
ACR based test	X			
Pregnancy test[7]	X			X
Assessment through inclusion/exclusion criteria	X	X		
Stoppage of pain reliever administration	X			
Confirmation of pain reliever administration stoppage		X		
Random assignment		X		
100mm Pain VAS assessment	X	X	X	X
K-WOMAC assessment		X	X	X
Patient Global Assessment			X	X
Adverse events			X	X
Confirmation of change in co-administered drugs[8]			X	X
Prescription of investigational product		X	X	
Rescue drug prescription and distribution of rescue drug log	X	X	X	
Survey on frequency of rescue drug usage		X	X	X

1	For subjects administered and treated with non-steroid anti-inflammatory pain reliever, osteoarthritis supplements, physical therapy, and oriental therapy(Acupuncture, cupping, moxibustion) within 2 weeks prior to screening must stop such administration and treatment for 14 days for a washout period and subjects without administration and treatment within the past 2 weeks can be administered with investigational products without having a washout period.	
3	It can be substituted with test results within 3 months from screening date.	
4	It can be substituted with test results within 4 weeks from screening date.	
	Hematology test	WBC, RBC, Hemoglobin, Hematocrit, Platelet count, WBC Diffcount
	Blood chemistry test	Na, K, Ca, Cl, BUN, Creatinine, Uric acid, Total bilirubin, Albumin, Total protein, Creatine Kinase, ALT, AST, r-GT, Alkaline phosphatase, glucose, Total cholesterol, Triglyceride
	Urine test	Protein, Glucose, Urobilinogen, WBC, RBC
5	Coagulation test	PT, aPTT<br>Performed during Visit 1.
6	Image knee AP. Lateral and it can be omitted if there are test results within past 4 weeks.	
7	Perform hCG test through urine for women of childbearing age.	
8	Medication of pain reliever 24 hours prior to the visit is restricted and visits can be rescheduled upon medication within visit window.	

1. INTRODUCTION

1.1 Background

Numerous non-steroid anti-inflammatory pain relievers are being developed and commercialized but they have the defect of having many side effects on the digestive system.

And (±)-(E)-2·[4-(2·oxocyclohexy lidene methyl)phenyl]propionic acid was developed as a result of two-sided evaluation from medicinal effect and effect on digestive system disorders by synthesizing numerous aromatic anhydrides and aromatic propionic acid derivatives to create an anti-inflammatory pain reliever strong anti-inflammatory, pain relief, and fever relief properties while minimizing digestive system disorders.

Safety and medicinal effect of Pelubiprofen were confirmed through previous clinical trials(Single/repetitive administration of main ingredient and metabolite, reproduction, occurrence, heredity, immunotoxicity, carcinogenesis, artery contraction effect in prenatal life, remedial effect, general pharmacology, ADME, etc.) and the advantages of Pelubiprofen emphasized from previous clinical trial results were: 1. Anti-inflammatory effect in Pelubiprofen compared to indomethacin, ketoprofen, naproxen was greater and Pelubiprofen especially exhibited strong anti-inflammatory effect and pain relief in the adjuvant arthritis test which highlighted its strong anti-inflammatory effect for chronic inflammations, 2. Pelubiprofen is quickly absorbed into the gastrointestinal tract which is a prodrug type pharmaceutical with satisfactory conversion rate and speed into active metabolite(trans-OH compound, unsaturated OH compound) with strong anti-inflammatory pain relief properties, 3. In terms of safety coefficient for effect on gastric mucous membrane regarding anti-inflammatory effect, pain relief, and fever relief(Dosage for 50% gastric mucous membrane disorder/50% rescue drug dosage/50% effective dosage), it resulted in large safety coefficients for all items especially with its safety coefficients being greater in the inflammation model(Adjuvant arthritis, carrageenan edema, etc.) compared to indomethacin, ketoprofen, naproxen which highlighted it as an extremely safe drug.

Daewon Pharmaceutical Co., Ltd. conducted a clinical trial on osteoarthritis

based on such study results. It reviewed the appropriate dosage through a two-phase clinical trial conducted at Gangnam Severance Hospital and Seoul Asan Medical Center. And it conducted a comparative clinical trial against the standard drug of diclofenac sodium which is known to have outstanding pain relieving effect among anti-inflammatory pain relievers through Gangnam Severance Hospital and 6 other institutions to complete the verification of safety and effectiveness.

Pelubiprofen was authorized for sale by KFDA in April of 2007 as effective drug for osteoarthritis based on domestic two-phase and three-phase clinical trial results as well as clinical trial results from Japan.

In 2009, clinical trials for addition of effect on backache were conducted through Soonchunhyang University Hospital and 9 other institutions to complete the verification of safety and effectiveness in the comparative clinical trial against aceclofenac tablets for KFDA approval in January 2011.

In 2010, clinical trials comparing the effectiveness and safety against that of celecoxib capsule in rheumarthritis patients were conducted through Seoul National University Hospital and 13 other institutions to receive additional approval from KFDA as of July 2012. Pelubi tablet(Pelubiprofen 30mg) as a result has secured various clinical effectiveness for controlling pain caused from osteoarthritis, backache, and rheumarthritis.

Daewon Pharmaceutical Co., Ltd. Also developed the Pelubi CR tablet(Pelubiprofen 45mg) which is taken twice per day to sustain effective drug concentration level for 12 hours equivalent to a slow release version of quick release drug Pelubi tablet(Pelubiprofen 30mg) which is taken 3 times per day in order to improve convenience for patients medicated with pain reliever for chronic pain. And it conducted a comparative clinical trial against Pelubi tablet on chronic backache patients in 2014. It was observed in the results of these clinical trials that Pelubi CR tablet(Pelubiprofen 45mg) exhibited similar pain relief effect compared to the traditional quick release tablet and the incidence rates of adverse event for the test group was 17.72%(14/79 people, 17 cases) while it was 17.50%(14/80 people, 20 cases) for the control group which was statistically insignificant. The company received approval for sale

from KFDA as Pelubi CR tablet being effective in terms of pain relief for osteoarthritis, backache, and rheumarthritis in April 2015 based on clinical trial results above.

Daewon Pharmaceutical Co., Ltd. planned this clinical trial to conduct a phase 4 clinical trial within the authorized area for Pelubi CR tablet(Pelubiprofen 45mg) to secure safety data and to confirm its clinical effectiveness. This clinical trial utilized aceclofenac tablet as control drug to confirm the safety and effectiveness of Pelubi CR tablet(Pelubiprofen 45mg) in patients with osteoarthritis of the knee.

2. PURPOSE AND HYPOTHESIS

The purpose of this clinical trial is to assess effect of pain relief and safety assessed by 100mm Pain VAS for each group after administering the Pelubi CR tablet and the aceclofenac tablet for 28 days to patients with osteoarthritis of the knee to prove that the effect of pain relief for Pelubi CR tablet is not inferior compared to that of aceclofenac tablet.

2.1 Primary Purpose

Comparison of treatment effectiveness between study drug and control drug regarding the effect of pain relief assessed by change in 100mm Pain VAS(Visual Analogue Scale) on the 28th day compared to baseline

2.2 Secondary Purpose

Comparison of treatment effectiveness between study drug and control drug assessed by changes in 100mm Pain VAS, K-WOMAC scale, and patient global assessment on the 14th day compared to baseline along with comparison of safety in terms of adverse events, vital signs, laboratory tests, and physical examinations

3. OVERALL DESIGN AND PLAN

3.1 Design

This clinical trial was designed as a multicenter, randomized, double-blinded, parallel, active-controlled, phase IV clinical trial.

Once the subject gives written consent to participate in the clinical trial, subjects who have been medicated with non-steroid anti-inflammatory pain reliever including narcotic analgesic, osteoarthritis supplements and treated with physical therapy and oriental therapy(Acupuncture, cupping, moxibustion) within 2 weeks prior to administration of clinical trial must undergo a washout period by stopping the administration and application of such treatments for 3~14 days prior to administration of investigational product. Screening period including the washout period cannot exceed 2 weeks.

Subjects who are not restricted in terms of prohibited medication and therapy prior to screening visit can skip the washout period if they meet the subject qualification criteria and be administered with investigational product.

Subjects who are qualified as the result of qualification examination results according to the selection criteria after completing the washout period are randomly assigned at the ratio of 1:1 to control group or test group. Randomly assigned subjects are medicated with study drug or control drug for 28 days and they are evaluated in terms of effectiveness and safety by visiting the testing institution on day 14 and 28 after medication.
frequency

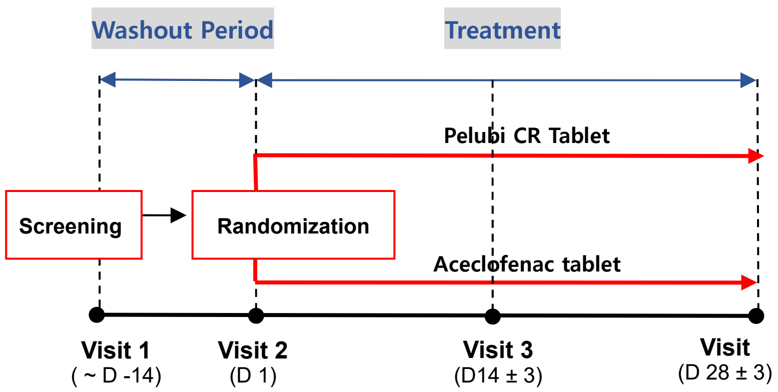

3.2 Number of Subjects and Basis of Calculation

3.2.1 Number of Subjects

95 Applicants qualified by inclusion and exclusion criteria were selected per group with a total of 190 applicants to secure 85 subjects that can be evaluated per group for effectiveness analysis for this clinical trial.

	Test Group	Control Group	Total Subjects
Number of cases for effectiveness assessment	85	85	170
Number of cases including 10% dropout rate	95	95	190

3.2.2 Basis of Calculation

The primary purpose of this clinical trial is to prove noninferiority of test group compared to control group within clinical tolerance limit regarding the effect of pain relief evaluated by 100mm Pain VAS compared to baseline after administering the study drug or control drug for 28 days.

This clinical trial inferred from the study results of aceclofenac tablet(Aceclofenac vs paracetamol in the management of symptomatic osteoarthritis of the knee : a double-blind 6-week randomized controlled trial. Osteoarthritis and Cartilage 2007;15(8):900-908) in order to calculate the number of subjects necessary to prove noninferiority for this clinical trial. As a result of clinical trial conducted on patients with osteoarthritis of the knee for

aceclofenac tablet in terms of change in 100mm Pain VAS and standard deviation was -18.34±24.86mm and the combined standard deviation for this clinical trial based on this result was established as ±24.86mm and clinical tolerance was established as -15mm which came from references[11].

The following assumptions and hypotheses were established in order to prove noninferiority within clinical tolerance limit in terms of change in test group compared to that of control group.

(1)Level of significance, α=0.025(One-sided test)
(2)Power of the test, 1-β=0.975
(3)Test group to control group ratio, λ=1
(4)Standard deviation between test and control groups, $\sigma_t=\sigma_c$=24.86m
(5)Clinical tolerance for noninferiority test, δ=-15mm
(6)Trace loss rate is assumed to be 10%(r=0.10)

Hypotheses of the noninferiority test for verification are as below.

$$H_0 : \mu_t - \mu_c \geq \delta$$
$$H_1 : \mu_t - \mu_c < \delta$$

The test variables required for the clinical trial are as below assuming (1)~(5) are true.

$$n_t = \frac{(\sigma_t^2 + \sigma_c^2)(Z_{1-\alpha} + Z_{1-\beta})^2}{\delta^2}$$

Number of subjects required per group according to equation above is 85 and it becomes 95 people per group considering the dropout rate of 10%.

3.3 Participant Clinical Trial Institutions

190 People(95 People per treatment group) will participate in this clinical trial with 9 institutions total in order to secure randomly assigned subjects. Refer to Attachment 3. Clinical Trial Performing Institutions and Investagors for more details.

3.4 Assignment of Administration Group

The investagor shall assign unique identification code for each subject in the order of registration.

Subjects qualified by inclusion and exclusion criteria will be randomly assigned according to random assignment plan and assigned unique random assignment number.

Assignment ratio per group for this clinical trial shall be 1:1 and this will not be disclosed in this clinical trial's plan.

When Group A is administered the study drug, Group B is administered the control drug and when Group A is administered the control drug, Group B is administered the study drug. Random assignment chart and drug for assigned groups will be disclosed after conclusion of clinical trial to maintain the blind.

Random assignment chart sequentially applies the random numbers generated from the randomization program of SAS system(Random number of A or B) from subject number 1 and it is generated prior to clinical trial through SAS V9.2 by a statistician.

Daewon Pharmaceutical Co., Ltd shall label the investigational product during packaging and transfer the investigational products to the managing pharmacist of corresponding clinical trial performing institutions.

4. METHOD AND PROCEDURE OF ASSESSMENT

4.1 Method of Assessment

4.1.1 Effectiveness Assessment

4.1.1.1 100mm Pain VAS

Subjective level of osteoarthritis of the knee pain felt by the subject will be indicated on the linear scale below using a 100mm line ranging from 'No pain'(0mm) to 'Most severe pain'(100mm) and the researcher shall review the indicated number and record the unit of mm.
*Actual subjects will use 100mm VAS scale with 20mm intervals.

No pain	Most severe pain
0	100

4.1.1.2 K-WOMAC(Korean version Western Ontario and McMaster Universities Osteoarthritis Index)

K-WOMAC survey(11.4) consists of 24 questions total over 3 areas with 5 questions on pain, 2 questions on stiffness, and 17 questions on physical function. Subjects will directly record the symptoms of the knee within 48 hours before visit through the 5-point Likert scale('None'(0), 'Slightly'(1), 'Mild'(2), 'Severe'(3), 'Very severe'(4)).

4.1.1.3 Patient Global Assessment

Subjective severity of disease and overall improvement in osteoarthritis of the knee will be evaluated using the following 5 point rating scale by the subject for final evaluation of effectiveness.

5 : Very good
4 : Good
3 : No change
2 : Poor
1 : Very poor

4.1.1.4 Frequency of Rescue drug Usage

Check for ratio and amount of medication among subjects by checking adminis tration of rescue drug distributed to subjects upon every visit excluding visit 1.

4.1.2 Safety Assessment

4.1.2.1 Adverse Reactions

Subjects shall be trained to voluntarily report on information regarding adverse events and the investagor shall check for adverse effects through interview a nd surveys conducted on regular and additional visits. Adverse event investiga tion includes occurrence and disappearance dates, severity and results of adv erse event, actions taken related to test/control drug, cause-and-effect relationship with test/control drug, name of substance suspected aside fr om test/control drug, treatment and details regarding adverse effect.

4.1.2.2 Pregnancy Test and Clinical Pathology Examination

Evaluation of clinical pathology examination shall be conducted according to th e clinical trial schedule at the laboratory of testing institution.

Clinical pathology examination is conducted on all subjects and evaluates over all health of the subject. Inclusion and exclusion criteria including pregnancy te st and clinical pathology examination must all be checked prior to administratio n of investigational product.

Additional tests are permitted by decision of clinical trial physician aside from t ests according to the schedule when necessary. However, tests can be omitte d if there are test results within 4 weeks from visit 1.

Test items include the following.

- Hematologic test : WBC, RBC, Hemoglobin, Hematocrit, Platelets count, WBC Diffco unt
- Blood chemistry test : Na, K, Ca, Cl, BUN, Creatinine, Uric acid, Total bilirubin, Albumin, Total protein, Creatine Kinase, ALT, AST, rGT, Alkaline phosphatas e, glucose, Total cholesterol, Triglyceride
- Blood coagulation test : PT, aPTT(Conducted only during visit 1)
- Urine test : Protein, Glucose, Urobilinogen, WBC, RBC

· Pregnancy test : Urine hCG (Only for women of childbearing age)

Criteria for reporting abnormal test results as adverse event is as below.

- When test results are relevant to associated symptoms
- When test results require additional diagnostic tests or medical/surgical trea tment
- When test results cause change in dosage of study drug, suspension of trial , additional treatment using significant co-administered drugs or non-medicated therapy
- When test results are considered to be an adverse event by the investagor or the client

Simple repetition of abnormal clinical pathology test without meeting any of the conditions above is not reported as an adverse event. There is no need to rep ort as an adverse event if the abnormal test result was caused by an error.

Women of childbearing age aside from subjects with confirmed sterilization op eration and menopause (Includes surgical menopause) shall be checked for pr egnancy through urine hCG test during visit 1 and 4.

4.1.2.3 Measurement of Vital Signs and Weight

Vital signs should be measured upon every visit and check for systolic/diastolic blood pressure and pulse in sitting position.

Weight should be measured upon every visit without jacket, shoes and with e mpty pockets and the scale used for measurement should be the same scale f or every visit if possible.

4.1.2.4 Physical Examination

Conduct physical examination according to the clinical trial schedule. Physical examination includes exterior, head, chest/lungs, heart, abdomen, urinary/repr oductive system, limbs, musculoskeletal system, nervous system, and lymphoi d organs.

Significant items discovered during screening should be recorded under

physical examination on the case report and significant items in terms of physical examination opinion meeting the definition of adverse event after administration of investigational product should be recorded under adverse events on the case report. However, occurrence of symptoms and signs that are not ideal prior to administration of investigational product shall be recorded under current medical history investigation on the case report.

4.1.2.5 ECG

ECG should be conducted for all subjects during visit 1 and 4 as 12-lead ECG and normal/abnormal test results as well as clinical significance for abnormal results should be recorded in the case report. Also, ECG may be con ducted selectively during the visit due to premature conclusion due to adverse effect by decision of the investagor when necessary. However, this test can be omitted if there are test results within 3 months from visit 1.

4.1.2.6 X-Ray Examination

X ray imaging shall be conducted on diseased joint area during visit 1. Test res ults must comply with ACR diagnosis criteria listed under Item 11.3 and there s hould be no opinion applicable for exclusion criteria. However, this test can be omitted if there are test results within 4 weeks from visit 1.

4.1.3 Other

4.1.3.1 Demographic Survey

Prior to clinical trial, describe the purpose and details of this clinical trial in deta il to subject, receive written consent, and assign screening number according t o sequence of written consent given followed by demographic survey.

Records should include written consent given, date of consent, gender, birthda y, age, etc.

4.1.3.2 Medical/Medication History

Investigate and record medical history and medication history of subject throug h interview and review of past diagnosis records. Details included in medical hi story and medication history investigations are as below.

For medical history, record past and current medical history including surgical history within past year prior to participation in clinical trial as well as hypersen sitivity, occurrence period (Year or month/year), and investagor opinion. Investi gate disease period for osteoarthritis of the knee in detail through interview wit h patient.

Medication history should include medication history and administration (Admi nistration method, dosage, administration period, etc.) within 4 weeks (2 Mont hs for hyaluronic acid and 3 months for steroid drugs).

Compare with confirmed details from the medication history during screening t o investigate any changes and record any changes in co-administered drugs in detail on the case report.

4.2 Procedure by Visit

4.2.1 Visit 1(Screening and Washout Period, 14 Days Prior to Trial)

Subjects selected for participation in this study shall be evaluated according to following sequence after listening to description of the study.

1) Describe trial procedure to subject and receive written consent form from subject prior to participation.
2) Assign screening number to subject in sequence of consent.
3) Survey and record demographic data and medical/medication history(Past and current history including surgeries).
4) Measure vital signs and weight and conduct physical examination.
5) Measure weight.
6) Conduct laboratory tests(Hematologic test, blood chemistry test, urine test, blood coagulation test).
7) Conduct pregnancy test for female subjects of childbearing age.
8) Perform ECG.
9) Conduct x-ray examination.
10) Evaluate according to ACR diagnosis criteria.
11) Evaluate according to inclusion/exclusion criteria.
12) Stop administration if subject is being regularly medicated with pain reliever and such and train on the washout period over 3~14 DAYS.

13) Evaluate 100mm Pain VAS.
14) Prescribe rescue drug.
15) Train on rescue drug log writing and distribute rescue drug log.
16) Schedule next visit.

4.2.2 Visit 2(Random Assignment and Administration of Investigational product, Day 1)

This visit takes place after washout period(3~14 days) for subjects administered pain relievers and such and the evaluations for this visit are as below.

1) Check for changes in medical/medication history compared to previous visit.
2) Check for washout period for pain reliever, etc.
3) Measure vital signs and weight and conduct physical examination.
4) Evaluate 100mm Pain VAS.
5) Combine all examination and evaluation results including clinical lab results up until this point and perform final evaluations whether qualified subjects according to inclusion/exclusion criteria have been selected.
6) Assign assignment number through random assignment.
7) Evaluate K-WOMAC.
8) Train on administration method and distribute investigational products.
9) Recover residual clinical trials from the subject and record residual quantity.
10) Distribute new rescue drug.
11) Train on rescue drug log writing and distribute new rescue drug log.
12) Schedule next visit.

4.2.3 Visit 3(Intermediary Visit, Day 14±3)

This visit takes place on day 14 after initial administration of investigational products and evaluates the following items.

1) Check for changes in co-administered drugs compared to previous visit.
2) Investigate for occurrence of adverse events and its details.
3) Measure vital signs and weight and conduct physical examination.
4) Evaluate 100mm Pain VAS.
5) Evaluate K-WOMAC.

6) Evaluate Patient Global Assessment.
7) Train on administration method and distribute investigational products.
8) Recover residual clinical trials from the subject and record residual quantity.
9) Distribute new rescue drug.
10) Train on rescue drug log writing and distribute new rescue drug log.
11) Schedule next visit.

4.2.4 Visit 4(End of Clinical Trial, Day 28±3)

This visit takes place on the last day of the study for final effectiveness evaluation of subjects administered investigational products for 28 days. Also, the following items are evaluated even if the clinical trial ends prematurely.

1) Check for changes in co-administered drugs compared to previous visit.
2) Investigate for occurrence of adverse events and its details.
3) Measure vital signs and weight and conduct physical examination.
4) Conduct laboratory tests(Hematologic test, blood chemistry test, urine test).
5) Conduct pregnancy test for female subjects of childbearing age.
6) Perform ECG.
7) Evaluate 100mm Pain VAS.
8) Evaluate K-WOMAC.
9) Evaluate Patient Global Assessment.
10) Recover residual clinical trials from the subject and record residual quantity.

4.2.5 Unscheduled Visit

Additional visits aside from scheduled visits may take place at the request of subject or subject's representative or by decision of the investagor when necessary.

5. SUBJECT SELECTION AND DROPOUT CRITERIA

5.1 Inclusion Criteria

Patients meeting the following conditions are selected as subjects.

1. Male and female patients between age 35~80
2. Patients diagnosed with osteoarthritis of the knee according to ACR diagnosis criteria with Kellgran & Lawrence stage I~III diagnosis on x-ray imaging
3. Patients evaluated with osteoarthritis to be stable for 3 months prior to clinical trial by clinical physician
4. Patient with at least 40mm on 100mm Pain VAS scale during visit 2
5. Patients who voluntarily gave consent in written form to participate in this clinical trial

5.2 Exclusion Criteria

Patients meeting following conditions are excluded from subjects.

1. Patient with secondary osteoarthritis
2. Patient suffering from disease that may affect the effectiveness assessment such as bursitis, tenosynovitis, fibromyalgia syndrome, rheumarthritis, and other inflammatory joint disease
3. Subject with confirmed with an ulcer or stomach disease through gastroscope
4. Patient with history of invasive knee joint operation, knee joint injury, arthroscopic joint operation within 12 months prior to screening visit or patient scheduled operation during clinical trial period
5. Patient with history of artificial joint surgery for knee joint
6. Patient with malignant tumor(However, patient without remission within 5 years after completion of treatment can be registered)
7. Patient with regular administration of psychoactive drug or narcotic analgesic which may affect sense of pain for at least 3 months
8. Subject administered with glenoid cavity injection or systemic injection(Oral administration or absorption of corticosteroid for 1 day exceeding 1500㎍) of steroid within 3 months prior to screening visit

9. Subject with hyaluronic acid injection for knee joint within 2 months prior to screening visit
10. Subject with intercurrent disease or medicated with co-administered drugs that are prohibits prescription of non-steroid anti-inflammatory pain reliever
11. Patient with history of asthma, hives, and allergic reaction against aspirin or other non-steroid anti-inflammatory pain reliever(COX-2 inhibitor included)
12. Patient requiring treatment for pain before and after CABG
13. Patient with inherited galactose intolerance, lapp deficiency, or glucose-galactose malabsorption
14. Patient with inflammatory bowel disease such as Crohn's disease or ulcerative colitis
15. Patient with severe renal disorder
16. Patient with severe liver disorder
17. Severe uncontrollable high blood pressure(Systolic blood pressure≥160mmHg, diastolic blood pressure≥100mmHg)
18. Patient with clinically significant systemic disease such as hematologic, heart(Congestive heart failure, ischemic heart disease, etc.), lung, nervous disease
19. Patient who does not consent for birth control* method permitted for this clinical trial among female subjects that may become pregnant during clinical trial period
    *Medically permitted birth control method: Condom, usage of intravenous and inserted contraceptive, installation of birth control device within the vagina, oral birth contraceptive
20. Subjects with dependency of drugs or alcohol
21. Other patients disqualified by the researcher

5.3 Dropout and Disqualification Criteria

Subject may stop participation at any time upon request or be dropped by the decision of the investagor or client for safety, behavioral, or administrative reasons at any time. The investagor shall question the subject regarding the grounds for dropout and return all investigational products not taken by the subject as well as requesting for a last visit and making maximum effort to perform follow-up observations for adverse events not resolved at the time

when applicable.

Instances when the subject may stop clinical trial are as below.

① When the subject or legal representative of subject withdraws consent for clinical trial participation
② Violation of inclusion/exclusion criteria
③ Violation of test plan outlined in the clinical trial plan
④ When there is an issue in administration of investigational product for subject
⑤ When subject cannot be traced
⑥ Instances where participation in clinical trial is deemed inappropriate by the investagor

5.4 Clinical Trial Compliance and Action for Plan Violation

Supervisor and instructor for this clinical trial shall understand and strictly perform the clinical trial according to the plan to prevent violations of clinical trial plan. The clinical trial supervisor shall conduct telephone monitoring and send out written notifications regarding future visiting hours as well as taking appropriate action for subjects for visitation for compliance with clinical trial administration and examination schedule. Violations of clinical trial plan which occur despite such efforts will be processed as below.

Critical violations of clinical trial plan will result in disqualification of corresponding subject from analysis(Excluding PPS) by principle and the conditions are as listed below.

① Lack of consent form
② Violation of inclusion/exclusion criteria
③ Administered with prohibited co-administered drugs during clinical trial period
④ Omission of major examination in the trial period

Other minor violations of clinical trial plan that are deemed to not affect other matters and interpretation of study results will be included in the PPS analysis after overall observation whether such violations have impacted the study

conducted by the investagor, client, monitor, and statistician when writing the conclusion report by listing the exact details and reason of violation or delay.

6. INVESTIGATIONAL PRODUCTS

6.1 Overview of Investigational products

6.1.1 Study drug

- Name: Pelubi CR tablet(Pelubiprofen 45mg)
- Property/Form: Round, light yellow slow release tablet with film coating
- Storage method: Sealed container without light exposure in room temperature(1~30℃)
- Expiration: 36 months
- Ingredients and content: 1 Tablet

Purpose	Substance	Spec.	Amount	Unit
Main ingredient	Pelubiprofen		45.0	mg

6.1.2 Control Drug

- Name: Airtal tablet(Aceclofenac 100mg)
- Property/Form: Coated white tablet
- Storage method: Sealed container without light exposure in room temperature
- Expiration: 36 months
- Ingredients and content: 1 Tablet

Purpose	Substance	Spec.	Amount	Unit
Main ingredient	Aceclofenac	EP	100.0	mg

6.1.3 Study drug Placebo

- Name: Pelubi CR tablet(Pelubiprofen) placebo
- Property/Form: Round, light yellow slow release tablet with film coating
- Storage method: Sealed container without light exposure in room temperature(1~30℃)
- Expiration: 36 months

6.1.4 Control Drug Placebo

- Name: Airtal tablet(Aceclofenac) placebo
- Property/Form: Coated white tablet

- Storage method: Sealed container without light exposure in room temperature
- Expiration: 36 months

6.1.5 Rescue drug

- Name: Tylenol ER tablet 650mg
- Property/Form: White rectangular film coated tablet
- Storage method: Sealed container without light exposure in room temperature
- Expiration: 36 months
- Ingredients and content:

Purpose	Substance	Spec.	Amount	Unit
Main ingredient	Acetaminophen		650.0	mg

6.2 Dosage, Administration Method, Administration Period

- Study drug : Oral administration of 1 Pelubi CR tablet(Pelubiprofen 45mg) after meal twice per day and take without breaking or chewing since it is a slow release tablet.
- Control drug : Oral administration of 1 Airtal tablet(Aceclofenac 100mg) after meal twice per day

Group	Morning	Evening
Test Group	1 Pelubi CR tablet + 1 Airtal tablet placebo	1 Pelubi CR tablet + 1 Airtal tablet placebo
Control Group	1 Pelubi CR tablet placebo + 1 Airtal tablet	1 Pelubi CR tablet placebo + 1 Airtal tablet

6.3 Production, Packaging, and Labeling of Investigational products

Investigational products are supplied to the managing pharmacist of clinical trial performing institution after the client manufactures or purchases the drugs.

Items listed on investigational products shall comply with Article 69 Clause 6 of the Regulation on Safety of Drugs, etc. and lists the following.

Sample production and maintenance methods for double-blind are as below.

① Separate storage : Study drugs and control drugs are stored separately by manufacturing number until labeling to prevent confusion.

② Labeling : Manufacture the label including the following details.

1. Indication of “For clinical trial”
2. Code name of product or generic name of main ingredient
3. Manufacturing number and expiration date or reinspection date
4. Storage method
5. Name and address of party approved for clinical trial plan
6. Indication of “Cannot use for purposes other than clinical trial”
7. Serial number: Recorded according to random assignment chart

③ Subdivided packaging : Drugs shall be subdivided and packaged by test group and control group according to 「3.4 Assignment of Administration Group」. Drugs shall be packaged sequentially to prevent confusion between test group and control group in terms of dosage and administration during subdivided packaging and stored separately until labeling. Investigational products(Study drug or control drug) shall be packaged by assignment code as below.

Group	Morning	Evening
Test Group	1 Pelubi CR tablet + 1 Airtal tablet placebo	1 Pelubi CR tablet + 1 Airtal tablet placebo
Control Group	1 Pelubi CR tablet placebo + 1 Airtal tablet	1 Pelubi CR tablet placebo + 1 Airtal tablet

④ Record of packaging and serial number: Drugs shall be packaged in quantity for 17 days(14 days+Extra 3 days; 34 packages) according to scheduled visits for each group and labeling listed in item ② above shall be performed for packaged drugs and each packaging shall be labeled as “Investigational product”.

⑤ Rescue drug shall be packaged individually.

6.4 Management of Investigational products

- Investigational products shall be stored at room temperature(1~30℃) and they cannot be used without instruction(Prescription) from trial supervisor and instructor.
- The client shall negotiate with the trial supervisor regarding investigational products and dispense the drugs to the managing pharmacist directly and keep a receipt. The investigational products at this time must be indicated “For clinical trial”.
- The managing pharmacist shall store and manage investigational products to prevent its usage aside from the purpose of clinical trial.
- The client shall check the quantity and storage of investigational products during clinical trial and take actions for the clinical trial to proceed appropriately.
- The client shall recover and dispose of unused investigational products upon suspension or conclusion of clinical trial or violation of clinical trial plan. The managing pharmacist at this time shall negotiate with the supervisor to return unused investigational products to the client and keep a return receipt.

6.5 Maintenance and Cancellation of Double-blind

This investigational product shall use double placebos for study drug and control drug for them to be impossible to distinguish by the exterior to maintain double-blind and history of serial number assignment for each group shall be managed by the trial supervisor in sealed state and not disclosed until the conclusion of clinical trial. Even when access to corresponding code is necessary due to occurrence of serious adverse event, the codes shall be managed in the form of double-blind envelope for each subject. However, the trial supervisor may consign such obligations to a person that is deemed appropriate among managing pharmacists or members of research team.

Cancellation of double-blind shall be considered case by case and it should only be considered for severe medical emergencies. Generally, blind must only be canceled when the information regarding the administration group impacts the treatment of subject. When cancellation of blind is deemed necessary by the trial supervisor or the client, the client or the trial supervisor shall make

contact to the other party for consent for cancellation of blind prior to cancellation of blind. Cancellation of blind in such instances shall be recorded and documented.

6.6 Compliance Assessment

Investigational product compliance shall be examined by the investagor in comparison of residual amount of investigational product brought by the subject upon every visit. Residual amount after administration must be returned to the pharmacy and recorded on dispense records. Remaining quantity after administration of rescue drug must also be returned to the pharmacy and recorded on dispense records.

$$\text{Drug compliance (\%)} = \frac{\text{Number of actual administrations}}{\text{Number of scheduled administrations}} \times 100$$

*Number of scheduled administrations: It is calculated by the number of administrations scheduled between the night of visit 2 or 3 and the morning of next visit.

6.7 Co-administered Drugs and Treatment

6.7.1 Permitted Drugs

Following drugs can be co-administered during clinical trial period.

1) Rescue drug(Acetaminophen below dosage of 1,950 mg per day) but administration 24 hours prior to visit is prohibited
2) Low dosage aspirin(Maximum daily dosage of 200mg) administered for prevention of cardiovascular disease must be administered without change in dosage until completion of clinical trial. However, administration 24 hours prior to visit is prohibited.
3) Co-administered drugs which were administered to the subject prior to participation in this clinical trial deemed to not impact the interpretation of

study results are permitted by decision of clinical trial physician.

4) Stomach system related drugs such as proton pump inhibitor and H2-blocker can be administered with prescription when adverse events related to stomach system occur or by decision of clinical trial physician.
5) Other drugs used daily for purpose of treatment for other disease and adverse events must be co-administered through consultation with attending physician.

Information(Name, purpose, dosage, administration period, etc.) on all drugs administered(Includes drugs for treatment of other disease and adverse events) shall be recorded in detail in case report for subject.

6.7.2 Restricted Drugs

Following drugs are restricted from co-administration during clinical trial period.

1) Surgical intervention
2) Steroid injection in glenoid cavity, orally administered steroid, hyaluronic acid injection
3) Muscle relaxant
4) Physical therapy and oriental therapy(Acupuncture, cupping, moxibustion) for pain relief, chiropractic therapy
5) Study drug, pain reliever excluding rescue drug, anti-inflammatory pain reliever(Locally spread solvent, patch included)
6) Psychoactive drug, narcotic analgesic, anticonvulsant
7) Sulfonylurea hypoglycemic agent(Tolbutamide, etc.) and new quinolone antibiotics(Enoxacin, etc.)
8) ACE inhibitor
9) Aspirin(When daily dosage exceeds 200mg)
10) Furosemide and thiazide diuretic(Hydrochlorothiazide, etc.)
11) Lithium
12) Methotraxate
13) Coumarin anticoagulant(Warfarin, etc.)
14) Osteoarthritis supplements such as glucosamine/chondroitin sulfate

Subject must stop participation in clinical trial when requiring use of restricted drugs for treatment during clinical trial period by decision of the investagor or

attending physician for treatment of other medical symptoms of the subject and its details must be recorded on the last page of case report.

7. EVALUATION VARIABLES AND PLAN FOR STATISTICAL ANALYSIS

7.1 Evaluation Variables

7.1.1 Primary Effectiveness Evaluation Variables

· Change in 100mm Pain VAS on 28th day compared to baseline

7.1.2 Secondary Effectiveness Evaluation Variables

1) Change in 100mm Pain VAS on 14th day compared to baseline
2) Change in K-WOMAC scale on 14th and 28th day compared to baseline
3) Patient Global Assessment
4) Frequency of rescue drug usage

7.1.3 Safety Evaluation Variables

1) Adverse reactions
2) Vital signs
3) Lab tests
4) Physical examination

7.2 Plan for Statistical Analysis

7.2.1 Definition of Analysis Sets

Analysis sets are defined as below.

(1) Safety analysis set: It consists of randomly assigned subjects who have been medicated with investigational products at least once.

(2) FAS (Full Analysis Set): It consists of subjects that yield data on primary effectiveness evaluation variables who have been medicated with investigational products at least once.

(3) PPS (Per-Protocol Set): It consists of subjects who completed the trial according to the clinical trial plan among subjects included in the FAS.

7.2.2 General Statistical Principles

Verification of all statistical significance shall be performed by a two-tail test

with significance level(α) of 5%. However, noninferiority will be verified using the confidence interval.

Main analysis of data regarding effectiveness from subjects of this clinical trial shall be performed through PPS followed by additional analysis of FAS by principle.

Analysis of data regarding safety shall be performed through the safety analysis set.

7.2.3 Demographic Data and Baseline Data Analysis

Mean, standard deviation, minimum and maximum values will be calculated for continues data in order to verify whether there is statistical difference between test group and control group regarding demographic and health statuses and comparison between the groups shall be performed through the t-test. Frequency between groups will be calculated and analyzed through the chi-square test or the Fisher's exact test in order to compare groups for categorical data.

Difference between groups regarding baseline observation values including demographic data of subjects will be observed in order to determine the pure treatment effect between test group and control group. Difference in demographic data or baseline observation values between test group and control group will result in such difference being considered as a disturbance factor should it be determined as being relevant to the effectiveness evaluation variables which is to be processed through subgroup analysis or being included in the effectiveness analysis as a covariate.

7.2.4 Effectiveness Analysis

The knee joint with greater value in terms of 100mm Pan VAS shall be selected as the subject knee joint if both knee joints of the subject meets the selection criteria. If the 100mm Pain VAS values are the same for both knee joints, the knee joint with a more severe arthritis as determined by the investagor shall be selected as the subject knee joint.

7.2.4.1 Primary Effectiveness Analysis

· Change in 100mm Pain VAS on 28th day compared to baseline

Calculate the upper limit(97.5% one-sided confidence interval) for 95% two-sided confidence interval for the difference in change of 100mm Pain VAS on the 28th day compared to baseline of test and control groups(Average change in test group - Average change in control group). If the upper limit of the confidence interval is below 15mm, then it shall be determined that the test group is not inferior compared to the control group. For this, configure the change in 100mm Pain VAS as dependent variable with the administration group as the random factor, and the baseline 100mm Pain VAS as the covariate to perform the covariance analysis. Suggest the calibrated average for the change in 100mm Pain VAS for each administration group based on this analysis and suggest the difference in calibrated means between the two group and its 95% confidence interval.

Additionally, mean, standard deviation, minimum and maximum values shall be suggested for the amount of 100mm Pain VAS measured at each visit for the test group and the control group and the change in 100mm Pain VAS on the 28th day compared to baseline.

7.2.4.2 Secondary Effectiveness Analysis

1) Change in 100mm Pain VAS on 14th day compared to baseline

Analysis through application of the same statistical method as the primary effectiveness evaluation variables.

2) Change in K-WOMAC scale on 14th and 28th day compared to baseline

Verify through the paired t-test for changes in total K-WOMAC score along with pain subscale, stiffness subscale, and physical function subscale on the 14th and the 28th days between test group and control group compared to the baseline.

Also, use the t-test to verify whether there is a difference between groups in terms of change on the 14th and the 28th day compared to baseline for each item.

3) Patient Global Assessment

Suggest the frequency and percentage of subjects evaluated as 'Very good(5)', 'Good(4)', 'No change(3)', 'Poor(2)', and 'Very poor(1)' on the 14th and the 28th day after investigational product administration and perform the chi-square test or the Fisher's exact test to see if there is a difference between groups.

If the overall assessment scores are 5 or 4, categorize as 'Response' and 'No response' if the scores are 3, 2, or 1 to suggest treatment response rates for each group. Perform the chi-square test or the Fisher's exact test to check for difference in treatment response rate between groups.

4) Frequency of rescue drug usage

Check the subjects medicated with rescue drugs each visit after administering the investigational products to suggest rescue drugs usage rate and perform the chi-square test or the Fisher's exact test to compare the rescue drug rates between each group followed by the t-test to check for difference in total usage amount between groups.

7.2.5 Safety Analysis

7.2.5.1 Adverse Reactions

All adverse reactions reported during trial period shall be charted and calculated for its incidence rate.

Ratio and frequency of subjects exhibiting adverse reactions in each group will be calculated and the adverse reaction incidence rates will be compared using the chi-square test or the Fisher's exact test. Same goes for drug reaction, serious adverse reaction, and adverse reaction causing the trial stoppage as incidence rate and frequency are suggested by administration group.

Occurring adverse reactions shall be summarized by administration group, body organ, and preferred term.

7.2.5.2 Clinical Laboratory Test

Suggest descriptive statistics for results observed in clinical laboratory tests before treatment(Screening) and after conclusion of study drug administration. Also, perform the paired t-test or Wilcoxon signed rank test to analyze whether

there is difference in change between the groups after conclusion of study drug administration compared to prior to treatment(Screening).

Also, suggest the frequency and ratio of subjects who were normal/clinically insignificant abnormal before treatment(Screening) that changed to clinically significant abnormal patients after conclusion of study drug administration.

7.2.5.3 Physical Examination and Vital Signs Examination Results

As for physical examination, compare the results of physical examination before treatment(Screening) and after conclusion of study drug administration and summarize the results into frequency and percentage in order to determine the change in normal/abnormal.

As for vital signs, suggest the descriptive statistics of results measured prior to treatment(Screening) and after conclusion of study drug administration. Also, perform the paired t-test or Wilcoxon signed rank test to analyze whether there is difference in change between the groups after conclusion of study drug administration compared to prior to treatment(Screening).

7.2.6 Processing of Dropout or Missing Values

The most recent data will be used for data analysis as if it was obtained at the corresponding point in time(Last Observation Carried Forward Method) should the subject is disqualified due to dropout or there is a missing value in data during FAS analysis before the clinical trial ends.

7.2.7 Scheduled Intermediary Analysis and Data Monitoring

Intermediary analysis and establishment of data monitoring committee were not planned for this clinical trial.

8. ADVERSE EVENTS

8.1 Definition of Adverse Events

(1) Adverse Events (AE)

It includes all harmful and unintended signs(Includes signs and anomalies in test results), symptoms, or diseases that occur in subjects administered with clinical trial drugs and it does not require a cause-and-effect relationship with the investigational product. Adverse events include but not limited to the following.

- Abnormal test results
- Clinically significant symptoms or signs
- Change in results of physical examinations
- Hypersensitivity
- Progression/Aggravation of existing disease

(2) Adverse Drug Reaction (ADR)

It refers to all harmful and unintended reactions in random dosages of investigational products where cause-and-effect relationship with investigational products cannot be denied.

(3) Unexpected Adverse Drug Reaction

It refers to difference in positive testing or hazard level of adverse drug reaction based on the drug substance related information provided through clinical trial data, attachment documents for drug substance, etc.

(4) Serious AE/ADR

It refers to one of the following among adverse events or adverse drug reactions occurring in random dosages of investigational products.

① When there is a death or danger of life

② When there is a need for hospitalization or extension of hospitalization period
③ When it causes a permanent or severe disability/performance decrease
⑤ When there is a malformation or anomaly in the embryo
⑥ Other medically serious circumstances

Even if it is not an event listed above but when an event that is considered to have significant impact on the well-being and health of patient by medical opinion occurs, determine whether it is considered as a serious adverse event through assigned physician and relevant experts and take appropriate action.

8.2 Collection and Record of Adverse Events

- Records of adverse events should be collected from after administration ofi nvestigational product until the final visit. All medical cases occurring prior t o administration of investigational product shall be recorded as current me dical history.
- Records of serious adverse events are collected after consent for participat ion in clinical trial is given and such events are all recorded as serious adv erse events even if they occur prior to administration of investigational prod uct.
- Adverse events should be reported including the name, sustained period(S tarting date/Disappearance date), severity, cause-and-effect relationship with investigational product, relevant actions, results, re medial treatment, and serious adverse events.
- Investagor should utilize standard medical terms to use name of overall dia gnosis or symptom rather than recording each symptom or sign when reco rding the adverse event.
- Adverse events occurring during clinical trial should be tracked until the ad verse event disappears or turns in stable results or until follow-up survey in subject fails.
- Only adverse events that are serious and related to investigational product should be reported for adverse events occurring after conclusion of clinica l trial.

8.3 Assessment of Adverse Events

8.3.1 Assessment of Severity

Severity of each adverse event and serious adverse reaction is assessed as below.

Grade 1 (Mild)	: Easily withstandable adverse event
Grade 2 (Moderate)	: Adverse event that disrupts daily life significantly
Grade 3 (Severe)	: Adverse event that makes daily life impossible

Definition of activities of daily life(ADL) is as below.
- All activities which occur throughout daily life to manage one's body
(Ex: Shower, dressing/undressing, eating, medicating, personal hygiene, personal care)

Adverse event or serious adverse reaction can all be assessed to be severe according to definition above but not all severe adverse events are serious adverse reactions.

8.3.2 Assessment of Cause and Effect Relationship

Relevance to investigational products for adverse events shall be assessed by the investagor by categories below.

① Definitely Related

- When there is evidence of this drug being administered
- When the administration of this drug is the most probable reason compared to other causes for the adverse effect
- When the adverse event disappears after stopping administration
- When re-administration(Rechallenge, perform only when possible) results test positive
- When the adverse effect is exhibiting symptoms consistent with information already known for this drug or drug of same group

② Probably Related

- When there is evidence of this drug being administered
- When chronological sequence of incidence and administration of this drug are logical
- When the administration of this drug is a more probable reason compared

to other causes for the adverse effect
- When the adverse event disappears after stopping administration

③ **Possibly Related**
- When there is evidence of this drug being administered
- When chronological sequence of incidence and administration of this drug are logical
- When the administration of this drug is about the same in terms of probability compared to other causes for the adverse effect
- When the adverse event disappears after stopping administration(When executed)

④ **Probably Not Related**
- When there is evidence of this drug being administered
- When there is another more probable cause for the adverse effect
- When results after stopping administration are negative or ambiguous (When executed)
- When the adverse event continues but ambiguous after stopping the administration of investigational product
-

⑤ **Definitely Not Related**
- When there is no evidence of this drug being administered
- When there is another highly probable reason for the adverse event
- When the adverse event does not disappear after stopping the administration of investigational product

⑥ **Unknown**

8.4 Report of Serious Adverse Events

All serious adverse events during clinical trial period must be reported via phone/fax to the client within 24 hours of acknowledgement by the investagor regardless of relevance to investigational product or its administration. Only serious adverse events related to investigational product shall be reported for adverse events that occur from the final investigational product administration until the 7th day after final administration.

In order to protect confidentiality of personal information of subjects when reporting serious adverse events to the client, the investagor must utilize subject identification codes instead of personal information of subjects such as their names, resident registration number, and address. All personal information aside from the identification code shall be redacted as necessary even when some copies of medical records are required.

Also, the investagor must perform a follow-up report if additional information regarding the serious adverse event has been acquired. The investagor must report regularly until the corresponding serious adverse event is concluded(Disappearance of corresponding adverse drug reaction or until follow-up report becomes impossible).

8.5 Report of Suspected Unexpected Serious Adverse Reaction(SUSAR) and Countermeasure

Clinical trial supervisor must determine continuance or stoppage of clinical trial by reporting to the institutional review board(IRB) upon occurrence of suspected unexpected serious adverse reaction(SUSAR) during clinical trial period and report to the Director of KFDA through the client within designated periods as listed below. Refer to the KFDA Clinical Trial Adverse Event Reporting Guidelines for further details.

① SUSAR which causes death or threatens life must be reported as quickly as possible to the Director of KFDA via phone/fax/document within 7 days from initial reporting date by the client and a complete report within 15 days of initial discovery is also required.

② All other SUSARs must be reported as quickly as possible within 15 days after client has been reported or notified.

Obligations of each party upon occurrence of “Suspected Unexpected Serious Adverse Reaction(SUSAR)” are as below. Clinical trial supervisor and instructor must focus on safety of subjects and take appropriate actions quickly upon occurrence of SUSAR to minimize the adverse reaction.

(1) Obligations of Clinical Trial Supervisor

Clinical trial supervisor must report any serious adverse drug reaction during clinical trial to the clinical trial review committee and client and stop the clinical trial for corresponding investigational product partially or entirely until further instructions.

(2) Obligations of Clinical Trial Instructor

Clinical trial instructor must report any critical adverse drug reaction during clinical trial to the clinical trial supervisor and client immediately.

(3) Obligations of Institutional Review Board

The institutional review board must take necessary actions such as order for stoppage for part or entire clinical trial should serious adverse drug reaction is observed through clinical trial supervisor.

(4) Obligations of Client

The client must submit the copy of report submitted by the clinical trial supervisor or clinical trial instructor regarding SUSAR to the Director of KFDA immediately and quickly notify the corresponding institutions should multiple institutions are performing the clinical trial.

8.6 Pregnancy

Pregnancy during clinical trial period is not considered as an adverse event and selective abortion(Abortion for treatment is not included) without complications or hospitalization for normal childbirth are also not considered as adverse events.

However, if a subject becomes pregnant during the clinical trial period(After administration of investigational product ~ Within 28 days of final administration), the subject shall be dropped from the clinical trial immediately and it shall be reported through the pregnancy report form within 24 hours of pregnancy discovery to the client. Investagor must perform follow-up report until childbirth for both the pregnant woman and the child even if the subject

stops participating in the clinical trial or after conclusion of clinical trial.
Severe complications of birthmother, natural miscarriage, ectopic pregnancy, stillbirth, death of newborn child, congenital malformation, and other events are considered to be severe adverse events and the investagor must report them accordingly.

9. ETHICS AND ADMINISTRATIVE PROCEDURE

9.1 Clinical Trial Plan Compliance

The investagor must conduct the clinical trial in compliance with the clinical trial plan. The clinical trial cannot be altered from the clinical trial plan unless elimination of immediate risk factors for the subject is necessary and any violations must be recorded with its details and grounds. Even if the investagor determines that the violation of the test plan can improve the performance of clinical trial, the investagor shall not execute the change until agreement from the client and the approval from the IRB(Includes the Director of KFDA if necessary) for the change are given.

9.2 Authorization and Revision of Clinical Trial Plan

When seeking authorization of clinical trial or revised clinical trial, authorization from the Institutional Review Board(IRB) is required for clinical trial plans and revised plans by stage as well as authorization from the Director of KFDA if necessary. Subjects cannot participate in the clinical trial prior to authorization.

9.3 Subject Consent Procedure

Subject description and consent forms can only be used after authorization from the Institutional Review Board(IRB). Consent from subject must be given according to ethics and pharmaceutical clinical trial management standards based on the Declaration of Helsinki. The investagor must describe the clinical trial to the subject(or the representative of the subject) prior to proceeding with all clinical trial related procedures and receive written consent from the subject. The investagor must store the original copy of signed consent form in the investagor files and provide the copy of written consent form along with description form explained to the subject(or the representative of the subject). Consent procedure for the patient must be recorded on the evidentiary materials.

When it is impossible for the subject to give consent, consent must be given through the representative of the subject. If neither the subject nor the representative of the subject is literate, an observer must be present for all procedures of giving consent. The subject or the subject's representative should sign the consent form if possible after agreeing to participating in the

clinical trial and the observer must sign on the consent form to prove that the information above was described and understood accurately.

If the subject description and consent forms are revised, they must be reapproved by the IRB and consent must be given by the subject(or the representative of the subject) already undergoing clinical procedure once again. The investagor at this time shall record the subject, date, and details of announcement on the evidentiary materials.

Also, vulnerable subjects(Ex: Hospital employees, school students, etc.) according to the opinion of IRB within testing institution may also be recruited and the clinical trial supervisor and instructor must comply with the following protective measures and plans along with the obligations as a investagor when registering 'Vulnerable subjects'. Recruitment of vulnerable subjects without approval from IRB of the testing institution cannot be registered.

[Protective measure and policy when registering 'Vulnerable subjects']
1) This clinical trial shall recruit employees within the hospital only through the method of posting a poster containing recruitment notice authorized by IRB in locations permitted by the hospital and employees that are directly related to the investagor in terms of chain of command shall not be recruited.
2) If the hospital employee wishes to participate through recruitment by posters within the hospital, sufficient description and time for consent procedure must be given according to KGCP and consent must be given according to the opinion of IRB in the corresponding institution through even more detailed description on importance of voluntary participation.
3) Subjects must be notified of contact information for IRB in order for them to independently discuss ethical issues or their rights regarding the clinical trial.

[Obligations of investagor when registering 'Vulnerable subjects']
1) Subjects should be able to freely and voluntarily decide on participation of clinical trial.
2) Clinical trial participation shall not be coerced in any form.
3) A subject can withdraw participation in clinical trial at any time.
4) There shall be no disadvantage related to participation of clinical trial.
5) Appropriate compensation as listed in the consent description form shall be

given like other subjects upon voluntary participation.
6) Employees directly related in chain of command shall not be recruited.

Subject consent description and written consent forms for this clinical trial can be found under Clinical Trial Plan Attachment 1.

9.4 Plan for Subject Safety and Protection

The investagor must perform the clinical trial keeping the rights and welfare of subjects in mind based on the Declaration of Helsinki and investagors participating in this clinical trial must perform the clinical trial in compliance with the Guidelines for Good Clinical Practice and the clinical trial plan. The investagor must assign sufficient time for each subject to strictly evaluate the qualifications and adverse events for subjects through interview and examinations.

Clinical trial supervisor must regularly report on adverse events, trial progress, status, and results to the client and the client shall regularly manage the progression of clinical trial.

9.5 Subject Consultation and Consultation Criteria after Clinical Trial

The investagor must allow subjects disqualified or not responding to treatment during clinical trials to receive other appropriate care and ensure appropriate substitute treatment if continued treatment is determined to be necessary for subject even after the conclusion of clinical trial.

9.6 Regulation for Victim Compensation

The client shall compensate the subject according to Clinical Trial Plan Attachment 2. Regulation on Victim Compensation for any damages directly caused by the investigational product as well as adverse events caused by investigational product and damages from the process of remedy for occurring adverse events.

9.7 Storage of Clinical Trial Related Documents and Records

9.7.1 Case Report and Evidentiary Materials

Data for this clinical trial will be collected using e-CRF. Data for case reports based on evidentiary materials must be consistent with its evidentiary materials and the investagor must ensure that all data entered into case report are accurate, conclusive, easy to understand, and timely.

The monitor shall compare the case report and the evidentiary material to notify and request for appropriate revision for any inconsistencies to the investagor. Only the investagor or the designated party may enter and revise data for case reports and evidentiary materials.

9.7.2 Access of Evidentiary Materials

Only the client, monitor, and auditor related to this trial may access the records of subjects for the purpose of monitoring, auditing, and progress management. The investagor may review or make copies of documents in order to verify the charts of subjects and case reports for the client, monitor, and auditor as the investagor enters into the agreement for this clinical trial. Such information must be confidential and be stored in appropriate facilities with appropriate regulations for management. The investagor must guarantee necessary support for the client and consigning institutions.

9.7.3 Storage of Clinical Trial Data

The investagor must store all data and records related to the clinical trial in a safe location and maintain security and store the data for 3 years from the final reporting date or stoppage date of clinical trial. Documents related to clinical trial shall be transferred to the security supervisor after completion of conclusion report and the client must be notified in advance should the investagor wishes to dispose of or relocate records related to the clinical trial.

9.7.4 Inspection and On-site Survey

Only the client or the party consigned by the client can perform quality assurance audit for this clinical trial in order to guarantee compliance with GCP and all other relevant regulations and the KFDA may conduct on-site surveys. The investagor must consent to audits and on-site surveys after receiving appropriate notification and permit the auditor or surveyor to directly access all documents related to the clinical trial and consent to assign time for discussion for all relevant matters and discoveries.

9.8 Confidentiality for Clinical Trial Documents and Subject Records

All clinical trial results and documents are confidential. Supervisors of the investagor, consigned institutions, and client are prohibited from disclosing clinical trial related information without written authorization from the client.

Records which contain information that can identify the subjects will be confidential and all documents related to the clinical trial including case reports will be recorded and identified with subject identification codes instead of names of subjects. Identity of subjects shall remain confidential even when the results of clinical trial are published.

9.9 Monitoring of Clinical Trial Performing Institution

Monitoring shall be conducted for protection of subject rights and welfare, confirmation of accuracy, completion, and verification possibility for all data through comparison of reported data and evidentiary materials related to clinical trial, and confirmation of compliance with Article 30 of the Regulation on Safety of Drugs, Etc. and Attachment 4. Pharmaceutical Clinical Trial Management Standards.

Monitor from CliPS Co., Ltd., clinical trial consigning institution shall monitor the clinical trial through regular testing institution visits and phone calls, assess the overall progression of clinical trial, and confirm the compliance with clinical trial plan and regulations. The monitor shall check the storage of original copies of subject records, case reports, pharmaceuticals management records, and research related data when visiting the institution and discuss with the investagor for any inconsistencies or issues discovered in the clinical trial records.

9.10 Stoppage of Clinical Trial

The client shall make corrections and take actions immediately should the consigned parties of testing institution, investagor, or client do not comply with GCP, clinical trial plan, and terms of contract. Confirmation of sustained violation along with other instances when registered goals expected to be completed are not complete and instances when effectiveness and safety information that may have significant impact on continuance of clinical trial is

discovered, the client may restrict the participation of clinical trial for the corresponding institution.

9.11 Clinical Trial Report and Publication

The client shall notify the results of clinical trial to the investagor after complete analysis of data from all clinical trial performing institutions to the investagor.

All data and results from this clinical trial are property of Daewon Pharmaceuticals Co., Ltd. which it reserves the right to announce the results of this clinical trial. The investagor cannot publish, announce, or disclose any information related to the results of this study without written consent from the client in advance and the same applies for the clinical trial instructor. All publication drafts and announcement drafts shall be provided to the client for discussion by the investagor in order to utilize only the accurate and verified data and announcement must be delayed until written authorization.

For multi-institution clinical trials, the investagor shall consent to not announcing the results of own institution or results of other institutions until the collective results from all clinical trial performing institutions are announced. However, exception is made for official request from the client and the clinical trial supervisor.

10. REFERENCES

1) 대원제약㈜. DW-330SR2 임상시험자자료집. 2013, Version 6

2) Jean-Yves Reginster, Janusz Badurski, Nicholas Bellamy, William Bensen, Roland Chapurlat, Xavier Chevalier, Claus Christiansen, Harry Genant, Federico Navarro, Evgeny Nasonov, Philip N Sambrook, Timothy D Spector, Cyrus Cooper. Efficacy and safety of strontium ranelate in the treatment of knee osteoarthritis_results of a double-blind, randomised placebo-controlled trial. Ann Rheum Dis 2013;72:179–186.
3) Estibaliz Loza. Systematic Review : Is There Contraindication to the Concomitant Use of Non-Steroidal Anti-Inflammatory Drugs and Steroids. Reumatol Clin. 2008;4(6):220-7
4) T. J. Schnitzer., J. R. P. Tesser, K. M. Cooper, R. D. Altman. A 4-week randomized study of acetaminophen extended-release vs rofecoxib in knee osteoarthritis. Osteoarthritis and Cartilage (2009) 17, 1e7
5) Supanimit Teekachunhatean, Puongtip Kunanusorn, Noppamas Rojanasthien, Kanit Sananpanich, Suwalee Pojchamarnwiputh, Sorasak Lhieochaiphunt, Sumalee Pruksakorn. Chinese herbal recipe versus diclofenac in symptomatic treatment of osteoarthritis of the knee: a randomized controlled trial [ISRCTN70292892]. BMC Complementary and Alternative Medicine 2004, 4:19
6) C. O. Bingham III, A. I. Sebba, B. R. Rubin, G. E. Ruoff, J. Kremer, S. Bird, S. S. Smugar, B. J. Fitzgerald, K. O'Brien and A. M. Tershakovec. Efficacy and safety of etoricoxib 30 mg and celecoxib 200 mg in the treatment of osteoarthritis in two identically designed, randomized, placebo-controlled, non-inferiority studies. Rheumatology 2007;46:496–507
7) A. Puopolo, J. A. Boice Ph., J. L. Fidelholtz, T. W. Littlejohn, P. Miranda, A. Berrocal, A. Ko., N. Cichanowitz. A randomized placebo-controlled trial comparing the efficacy of etoricoxib 30 mg and ibuprofen 2400 mg for the treatment of patients with osteoarthritis. Osteoarthritis and Cartilage 2007;15:1348-1356
8) MARC C. HOCHBERG, ROY D. ALTMAN, KARINE TOUPIN APRIL, MARIA BENKHALTI, GORDON GUYATT, JESSIE MCGOWAN, TANVEER TOWHEED, VIVIAN WELCH, GEORGE WELLS, PETER TUGWELL. American College of Rheumatology 2012 Recommendations for the Use of

Nonpharmacologic and Pharmacologic Therapies in Osteoarthritis of the Hand, Hip, and Knee. Arthritis Care & Research 2012;64(4):465–474
9) 윤종현. 골관절염의 최신지견 대한내과학회지. 2012;82(12):170-174
10) E. Batlle-Gualda, J. Roma´n Ivorra, E. Martı´n-Mola, J. Carbonell Abello, L. F. Linares Ferrando, J. Tornero Molina, A. Raber Be´ jar, J. Fortea Busquets. Aceclofenac vs paracetamol in the management of symptomatic osteoarthritis of the knee: a double-blind 6-week randomized controlled trial. Osteoarthritis and Cartilage (2007) doi:10.1016/j.joca.2007.02.008
11) Young-Wan Moon, Seung-Baik Kang, Tae-Kyun Kim, Myung-Chul Lee. Efficacy and Safety of Aceclofenac Controlled Release in Patients with Knee Osteoarthritis: A 4-week, Multicenter, Randomized, Comparative Clinical Study. Knee Surg Relat Res 2014;26(1):33-42
12) Anil Pareek, Nitin Chandurkar, Anil Gupta, Ashish Sirsikar, Bhavik Dalal, Bhavesh Jesalpura, Anoop Mehrotra, and Arunangshu Mukherjee, Efficacy and Safety of Aceclofenac-CR and Aceclofenac in the Treatment of Knee Osteoarthritis: A 6-Week, Comparative, Randomized, Multicentric, Double-Blind Study. The Journal of Pain, Vol 12, No 5 (May), 2011:546-553

11. APPENDIX

11.1 Expected Adverse Reactions and Use Precaution for Investigational product

11.1.1 Adverse Reactions and Use Precaution for Pelubi CR Tablet

1. Warning

(1) A person regularly drinking alcohol in excess of three glasses per day who is medicating with this drug and other fever and pain reliever must consult with a physician or a pharmacist. Medication of this drug for such person may cause gastrointestinal hemorrhage.

(2) Cardiovascular risk: Non-steroid anti-inflammatory pain relievers including this drug may increase the risk of severe cardiovascular blood clots, myocardial infarction, and cerebral stroke and such instances may be critical. Such risks may increase depending on period of administration. It may be great risk to patients with cardiovascular disease or risk factors for cardiovascular diseases.

(3) Doctor and patient should monitor occurrence of such cardiovascular symptoms with caution and the same applies even if the patient has no medical history of cardiovascular disease. The patient should be aware of actions to be taken upon occurrence of such critical cardiovascular symptoms and signs in advance.

(4) Gastrointestinal risk: Non-steroid anti-inflammatory pain relievers including this drug may increase risk of stomach or intestinal hemorrhage along with severe gastrointestinal adverse reaction including ulcer and perforation which could be fatal. Such adverse reactions may occur during administration period without warning symptoms. Risk of severe gastrointestinal adverse reaction may be greater for elderlies.

(5) Longer administration period may increase the risk of critical gastrointestinal adverse reaction but such risk is not eliminated for short term administration.

(6) Gastrointestinal ulcer along with symptoms and signs of hemorrhage should be monitored with caution during administration of this drug and additional evaluation and treatment must be performed immediately when severe gastrointestinal adverse reaction is suspected. Stopping

administration of non-steroid anti-inflammatory pain reliever until the severe gastrointestinal adverse reaction disappears completely could be a treatment method. Other substitute treatment drugs not related to non-steroid anti-inflammatory pain reliever should be considered for high risk group patients.

2. Do not administer to following patients

(1) Patient with peptic ulcer
(2) Patient with severely abnormal blood levels
(3) Patient with severe liver disorder
(4) Patient with severe renal disorder
(5) Patient with severe cardiac insufficiency
(6) Patient with severe high blood pressure
(7) Patient with hypersensitivity to substance of this drug
(8) Patient exhibiting asthma, hives, allergic reactions, and such medical history towards aspirin and other non-steroid anti-inflammatory pain reliever(Includes COX-2 inhibitor)(Critical anaphylactoid reactions have been rarely reported after administration of non-steroid anti-inflammatory pain reliever in such patients)
(9) Treatment for pain before and after CABG
(10) Breastfeeding woman
(11) This drug contains lactose and it should not be administered to patients with inherited galactose intolerance, lapp deficiency, or glucose-galactose absorption disorder.

3. Administer with caution to following patients

(1) Patient with history of peptic ulcer
(2) Patient with abnormal blood levels of such medical history
(3) Patient with tendency to hemorrhage(May exhibit platelet function disorder)
(4) Patient with liver disorder of such medical history
(5) Patient with renal disorder or such medical history
(6) Patient with heart disorder
(7) Patient with high blood pressure
(8) Patient with medical history of hypersensitivity
(9) Patient with bronchial asthma
(10) Patient with SLE and MCTD

(11) Patient with ulcerative colitis
(12) Patient with Chron's disease
(13) Elderlies and children

4. Adverse Reactions
4.1 General Definition of Adverse Reactions for Pelubiprofen
(1) Group administered with this drug in the clinical trial comparing against placebo or active substance on osteoarthritis, backache, and rheumarthritis patients consisted of 846 subjects total(59 Subjects for 30mg/day, 59 subjects for 60mg/day, 339 subjects for 90mg/day, 312 subjects for 120mg/day). 210 Subjects(24.9%) reported adverse events among 846 subjects for this group. Adverse event by organ was most frequent for digestive system(18.7%, 159 subjects) and whole system(8.1%, 69 subjects). Adverse events reported above 2% in terms of frequency were edema(4.6%), stomachache, heartburn, indigestion, nausea/vomit, and upper respiratory tract infection.
(2) Adverse events reported in clinical trial were listed in the descending order of frequency by body part in Chart 1 below. Cause-and-effect relationship with this drug was not taken into consideration. Frequency is as following: Very common(≥ 1/10), common (≥ 1/100, < 1/10), sometimes(≥ 1/1,000, < 1/100), rarely (≥ 1/10,000, < 1/1,000).

[Chart 1] Adverse Reactions Reported in Administration Group(846 people total) for this Drug During Clinical Trial

System	Frequency	
	Common	Sometimes
Peripheral nervous system	Headache, dizziness	Drowsiness, confusion, heavy head feeling, insomnia
Sensory system		Rubefaction, tinnitus, itch, dry eye, hives, visual impairment, eczema, dysaesthesia, vitreous floaters, dry skin, hair loss
Digestive system	Stomachache, heartburn, nausea/vomit, indigestion, abdominal discomfort, diarrhea	Constipation, appetite loss, abdominal inflation, glossitis, dry mouth, oral discomfort, stomatitis, appetite increase

Circulatory system		Chest pain, heart palpitation, blood pressure drop, cardiac infarction, myocardial ischemia, high blood pressure
Respiratory system		Dyspnoea, cough, nosebleed, laryngoxerosis
Urogenital system		Oliguria, urine decrease, colpitis, colporrhagia
Musculoskeletal system		Muscleache, backache, back pain, arthrosis, fracture
Whole system	Edema, facial edema	Fatigue, weight increase, chills, fever, chest discomfort, paleness, weight loss, pain
Infection	Upper respiratory tract infection	Viral infection
Lab tests		Blood urea nitrogen increase, white blood cell count decrease, high blood cholesterol level, liver enzyme level increase

(3) Investigation Results After Domestic Commercialization

① Results of usage performance survey on 3,381 survey participants over 6 years for re-evaluation in Korea reported incidence rate of 2.29%(77 people, 90 cases) regardless of cause-and-effect relationship and drug toxicity with cause-and-effect relationship with this drug among them was 1.54%(52 people, 62 cases).

As for reported adverse drug reactions, indigestion was highest at 0.71%(24 people, 24 cases) followed by edema at 0.35%(12 people, 12 cases), heartburn at 0.24%(8 people, 8 cases), stomachache at 0.18%(6 people, 6 cases), nausea at 0.09%(3 people, 3 cases) as well as appetite loss, constipation, dry mouth, hematochezia, facial edema, dizziness, muscleache, pollakisuria, and skin rash each respectively at 0.03%(1 person, 1 case). SUSAR which was not observed prior to commercialization were 'hematochezia' and 'skin rash' which were both respectively at 0.03%(1 person, 1 case).

② As a result of assessing the reported cases of side effects for re-

evaluation and voluntary side effect report data in Korea for this drug with all reported cases of side effects for all pharmaceuticals permitted for sale in Korea at conclusion of re-evaluation, the following side effects have been newly confirmed to be reported significantly more in terms of statistics for this drug compared to all other pharmaceuticals. However, these results do not signify proof of cause-and-effect relationship between the corresponding substance and the following cases of side effects.

- Musculoskeletal system: Bone pain

4.2 Adverse Reactions of Pelubi CR Tablet

(1) Adverse events were reported in 28 subjects total in randomly assigned, double-blind, activity comparison(Pelubiprofen tablet), parallel group clinical trial(n=159) performed on patients with backache and it occurred for 14 subjects among 79 subjects in the administration group for this drug(17.72%, 17 cases) while it was 14 subjects among 80 subjects(17.50%, 20 cases). 1 Case of severe adverse event (Humerus fracture) was reported but it was determined to be irrelevant to the study drug. Frequently exhibited adverse event in the administration group included indigestion, blood CPK level increase, and edema. Also, analysis of incidence rate for adverse drug reaction which includes cause-and-effect relationship with the study drug, only 10 subjects(12.66%. 12 cases) were reported for the administration group of this drug while 11 subjects(13.75%, 13 cases) were reported for the control group.

(2) Adverse events reported in administration group and control group for the clinical trial were listed as below in Chart 2.

[Chart 2] Manifestation by Type of Adverse Reaction

	Test Group(n=79) Pelubiprofen Slow Release Tablet			Control Group(n=80) Pelubiprofen Tablet		
	Incidence Rate		Number of Cases	Incidence Rate		Number of Cases
	n	(%)	n	n	(%)	n
Digestive system	6	(7.59)	6	8	(10.00)	9
Indigestion	4	(5.06)	4	5	(6.25)	5
Constipation	1	(1.27)	1	2	(2.50)	2
Nausea	1	(1.27)	1	1	(1.25)	1
Stomach ulcer	0	(0.00)	0	1	(1.25)	1
Lab tests	4	(5.06)	4	2	(2.50)	3
Blood CPK level increase	4	(5.06)	4	0	(0.00)	0
Blood triglyceride level increase	0	(0.00)	0	2	(2.50)	2
GGT increase	0	(0.00)	0	1	(1.25)	1
Status of whole system and administered area	3	(3.80)	3	2	(2.50)	2
Anasarca	3	(3.80)	3	1	(1.25)	1
Facial edema	0	(0.00)	0	1	(1.25)	1
Infection	1	(1.27)	1	3	(3.75)	3
Cystitis	0	(0.00)	0	1	(1.25)	1
Mycosis	0	(0.00)	0	1	(1.25)	1
Sore throat	0	(0.00)	0	1	(1.25)	1
Urinary tract infection	1	(1.27)	1	0	(0.00)	0
Skin and subdermal tissue disorder	1	(1.27)	1	1	(1.25)	1
Eczema	0	(0.00)	0	1	(1.25)	1
Itch	1	(1.27)	1	0	(0.00)	0
Circulatory system	0	(0.00)	0	1	(1.25)	1
Heart palpitation	0	(0.00)	0	1	(1.25)	1
Injury, addition, complications due to procedure	1	(1.27)	1	0	(0.00)	0
Humerus fracture	1	(1.27)	1	0	(0.00)	0
Renal and urinary system disorder	0	(0.00)	0	1	(1.25)	1
Pollakisuria	0	(0.00)	0	1	(1.25)	1
Respiratory, chest, mediastinal disorders	1	(1.27)	1	0	(0.00)	0
Cough	1	(1.27)	1	0	(0.00)	0

5. General Caution

(1)Consider the potential risk and benefit of this drug and other substitute treatment method before administering this drug. This drug should be administered at minimum effective dosage over the shortest period possible consistent with purpose of treatment for each patient.

(2)Keep in mind that treatment by anti-inflammatory pain reliever is not a causal treatment but a symptomatic treatment.

(3)Consider the following when using for chronic disease.

① Patients administered non-steroid anti-inflammatory pain reliever long term must undergo regular CBC and physicochemical tests. Stop the administration of this drug should clinical symptoms related to liver or renal diseases or systemic signs(Ex: Acidophilia, hives) is observed or if liver function test or renal function test results continue to be abnormal or worsen.

② Consider treatment methods aside from drug treatment.

(4)Consider the following when using for acute disease.

① Medicate considering acute pain and fever severity.

② Avoid long term administration of same drug by principle.

③ Perform causal treatment if applicable.

(5)Patients should be observed sufficiently and monitored for occurrence of adverse reaction. Patients may exhibit excessive body temperature drop, collapse, limb ischemia, and other symptoms so monitor the status with caution especially for children and elderlies or wasting disease patients accompanied by high fever.

(6)Other symptoms and signs of inflammation may become unapparent due to the nature of this drug's pharmacological properties thus delaying the diagnosis of infectious complications through pain and noninfectious conditions.

(7)Adverse gastrointestinal reaction: Extreme caution in prescription of non-steroid anti-inflammatory pain relievers including this drug should be exercised for patient with ulcerative disease or history of gastrointestinal hemorrhage. Patient with digestive ulcer disease and/or history of gastrointestinal hemorrhage is exposed to risk of gastrointestinal hemorrhage more than 10 times upon administration of non-steroid anti-inflammatory pain reliever compared to a patient without such risk factor. Other risk factors which increase the risk of gastrointestinal hemorrhage

are co-administration with oral corticosteroid or anticoagulant, long term use of non-steroid anti-inflammatory pain reliever with alcohol consumption, old age, and weak physical state. Most of voluntary reports regarding fatal gastrointestinal reactions come from elderlies and physically weak people so administration of this drug for such patients should be cautious.

(8)High blood pressure: Non-steroid anti-inflammatory pain relievers including this drug may cause high blood pressure or aggravate existing high blood pressure and such may result in increase in occurrence of cardiovascular failures. Patient administered thiazide diuretic or loop diuretic whom are medicated with non-steroid anti-inflammatory pain reliever may experience reduced reaction from such treatment. Non-steroid anti-inflammatory pain relievers including this drug must be administered with caution for high blood pressure patients. Blood pressure must be closely monitored during early stage of administration and throughout the administration period for this drug.

(9)Congestive heart failure and edema: Fluid retention and edema were observed in some patients medicated with this drug and other non-steroid anti-inflammatory pain reliever. This drug should be administered with caution for patients with fluid retention or cardiac insufficiency.

(10)Long term administration of non-steroid anti-inflammatory pain reliever may cause renal papillary necrosis or other renal damage. Also, special caution is required for cardiac insufficiency patients, renal function disorder patients, liver function disorder patients, patients administered with diuretic or ACE inhibitor, and elderlies since the role of prostaglandin is critical for maintaining RBF. Most patients should recover to state before treatment once administration is suspended.

(11)Progressing renal disease: There are no controlled clinical trials regarding usage of this drug on patients with progressed renal disease. Therefore, administration of this drug is not recommended for patients with progressed renal disease. Renal function of the patient should be monitored closely if administration of this drug is necessary.

(12)Administration of non-steroid anti-inflammatory pain relievers including this drug may result in elevated liver function levels. Such abnormal test results may get worse or remain the same or even be temporary as the treatment continues. Also, severe adverse reactions related to the liver including jaundice, fatal fulminant hepatic failure, hepatic necrosis, and hepatic

insufficiency(Some can be fatal) were rarely reported for administration of non-steroid anti-inflammatory pain relievers including this drug.

Patient with symptoms and/or signs of abnormal liver function or patient with abnormal liver function test results should be observed carefully throughout the administration period for aggravation of liver function abnormality and administration of this drug should be stopped when clinical symptoms or systemic signs(Ex: Acidophilia, hives) related to liver disease occur.

(13)Administration of non-steroid anti-inflammatory pain relievers including this drug may cause anemia so patients must be tested for hemoglobin count or HCT count when they exhibit symptoms or signs of anemia due to long term administration of this drug.

Non-steroid anti-inflammatory pain relievers suppress platelet aggregation and it has been confirmed to extend hemorrhage time in some patients. Unlike aspirin, impact of this drug on platelet function is comparatively small, short in duration, and reversible. Patients that may be negatively affected due to change in platelet function from co-administration of anticoagulant or with coagulation related disease should be monitored with caution after administration.

(14)Anaphylactoid reaction: Anaphylactoid reaction may occur in patients without previous exposure to the substance like other non-steroid anti-inflammatory pain reliever. Such complex symptom may or may not accompany nasal polyp after administration of aspirin or other non-steroid anti-inflammatory pain reliever or occur in asthma patients exhibiting symptoms of potentially fatal bronchospasm. First aid should be administered when such anaphylactoid reaction is observed.

(15)Skin reaction: This drug may cause serious adverse skin reaction such as deciduous dermatitis, Stevens-Johnson syndrome, and toxic epidermal necrolysis and they may be fatal. Such serious adverse reactions may occur without warning symptoms. Most of the time, such reactions occur within 1 month of initial administration. Patients should be aware of serious skin symptoms and signs and stop administration of this drug when skin rashes or other initial symptoms and signs of hypersensitivity occur.

(16)Some asthma patients may be sensitive to aspirin. Use of aspirin for aspirin sensitive asthma patient may be result in severe bronchospasm

which may be fatal. Cross-reaction including bronchospasm between aspirin and other non-steroid anti-inflammatory pain relievers in such aspirin sensitive patients has been reported. Therefore, this drug should not be administered to such aspirin sensitive patients and be administered with caution for asthma patients.

(17)This drug cannot substitute corticoid agents or be used as a drug for treating corticoid deficiency. Sudden stoppage in corticosteroid administration may cause aggravation of corticosteroid-responsive disease. Administration of this drug for a patient administered with corticosteroid long term requires gradual reduction of dosage.

(18)Patients experiencing other central nervous disorders including drowsiness, dizziness, and visual impairment should avoid driving or handling machinery.

6. Interaction

(1) Avoid co-administering with other anti-inflammatory pain relievers.

(2) Administration of this drug shall be performed with caution since it may increase the effect of the following drugs : Sulfonylurea hypoglycemic agent(Tolbutamide, etc.), new quinolone antibiotics(Enoxacin, etc.)

(3) ACE inhibitor: There are reports of reduced antihypertensive effect for ACE inhibitor due to non-steroid anti-inflammatory pain reliever so such interaction should be kept in mind for co-administration with ACE inhibitor.

(4) Aspirin : There is no consistent evidence that co-administration with aspirin reduces the risk of severe cardiovascular thrombus related to use of non-steroid anti-inflammatory pain reliever. Co-administration of this drug and aspirin may increase the risk of severe adverse gastrointestinal reaction like other non-steroid anti-inflammatory pain reliever so co-administration of these two drugs is generally not recommended.

(5) Furosemide and thiazide diuretics(Hydrochlorothiazide, etc.): It was confirmed that the natriuresis effect of furosemide and thiazide diuretics may decrease in some patients due to prostaglandin synthesis inhibitor effect from this drug on the kidney after clinical trial and follow-up study after commercialization. Co-administration of such substances and non-steroid anti-inflammatory pain reliever requires close observation for signs of renal failure.

(6) Lithium : Non-steroid anti-inflammatory pain reliever increases blood

serum lithium level and decrease of renal clearance rate of lithium due to inhibition of prostaglandin synthesis by the kidney. Therefore, signs for lithium toxicity should be monitored when co-administering lithium and non-steroid anti-inflammatory pain reliever.

(7) Methotrexate: Co-administration with non-steroid anti-inflammatory pain reliever may increase the toxicity of methotrexate so proceed with caution when co-administering both substances.

(8) Coumarin anticoagulant(Warfarin, etc.) : Warfarin and non-steroid anti-inflammatory pain reliever may have elevated effect regarding gastrointestinal hemorrhage so patient using both substances may be more exposed to risk of severe gastrointestinal hemorrhage compared to a patient administered with just a single substance. Such effect may be increased upon co-administration with coumarin anticoagulant so administration must be performed with caution.

7. Administration for pregnant women

(1) Embryo toxicity(Increase in mortality of embryo in high dosages) is being reported in animal tests and safety for pregnant women is not established so it is not recommended to administer this drug to a pregnant woman or a woman who may be pregnant.

(2) There is no clinical data on administration of this drug for pregnant women. Administration of this drug may prematurely shut down the arterial tube of the embryo towards late stage of pregnancy like other non-steroid anti-inflammatory pain reliever so administration of this drug should be especially avoided during late stage of pregnancy.

(3) There are reports of PEC occurring for other fever and pain relieving antiphlogistics.

(4) Test on rats for this drug revealed increased frequency of dystocia like other drugs that inhibit synthesis of prostaglandin as well as delay in childbirth and decrease in survival of newborn.

8. Administration for breastfeeding women

Lactic transfer has been reported in animal test(Rat) so this drug should not be administered while breastfeeding.

9. Administration for children

Safety for children has not been established.

10. Administration for elderlies

Adverse events tend to occur easily among elderlies so administration should begin from small dosage and be administered only with minimum dosage necessary and patient status should be observed with caution regarding administration interval.

11. Storage and handling precaution

(1) Keep out of children's reach
(2) Changing container may cause accidents and it is not recommended for quality maintenance

12. Other

(1) Genotoxicity: This drug did not exhibit mutagenicity as a result of ames assay and micronucleus test. However, it did test positive on chromosome anomaly test in presence and absence of metabolic activation system using mammal culture cell and 20% of the cells tested positive for chromosome anomaly with concentration level between 60~350 ㎍/ml.
(2) Reproductive/Developmental toxicity: Reproductive toxicity test using animals resulted in observation of digestive organ disorder in the embryo development test using rabbits when administering 100mg/kg/day and decrease in number of surviving embryos was observed at dosage of 300mg/kg/day. Dosage of 3mg/kg/day in rat pre-birth and post-birth development and parent function tests resulted in increase of mortality and the number of rats born decreased significantly.

11.1.2 Adverse Reactions and Use Precaution for Airtal Tablet

1. Warning

(1) A person regularly drinking alcohol in excess of three glasses per day who is medicating with this drug and other fever and pain reliever must consult with a physician or a pharmacist. Medication of this drug for such person may cause gastrointestinal hemorrhage.
(2) Cardiovascular risk: Non-steroid anti-inflammatory pain relievers including this drug may increase the risk of severe cardiovascular blood clots,

myocardial infarction, and cerebral stroke and such instances may be critical. Such risks may increase depending on period of administration. It may be great risk to patients with cardiovascular disease or risk factors for cardiovascular diseases.

Doctor and patient should monitor occurrence of such cardiovascular symptoms with caution and the same applies even if the patient has no medical history of cardiovascular disease. The patient should be aware of actions to be taken upon occurrence of such critical cardiovascular symptoms and signs in advance.

(3) Gastrointestinal risk: Non-steroid anti-inflammatory pain relievers including this drug may increase risk of stomach or intestinal hemorrhage along with severe gastrointestinal adverse reaction including ulcer and perforation which could be fatal. Such adverse reactions may occur during administration period without warning symptoms. Risk of severe gastrointestinal adverse reaction may be greater for elderlies.

Longer administration period may increase the risk of critical gastrointestinal adverse reaction but such risk is not eliminated for short term administration.

Gastrointestinal ulcer along with symptoms and signs of hemorrhage should be monitored with caution during administration of this drug and additional evaluation and treatment must be performed immediately when severe gastrointestinal adverse reaction is suspected. Stopping administration of non-steroid anti-inflammatory pain reliever until the severe gastrointestinal adverse reaction disappears completely could be a treatment method. Other substitute treatment drugs not related to non-steroid anti-inflammatory pain reliever should be considered for high risk group patients.

2. Do not administer to following patients

(1) Patient with active peptic ulcer/hemorrhage or such medical history

(2) Patient with hypersensitivity to this drug or its ingredients or other drugs(Diclofenac) of same group

(3) Patient exhibiting asthma, hives, allergic reactions, and such medical history towards aspirin and other non-steroid anti-inflammatory pain reliever(Includes COX-2 inhibitor)(Critical anaphylactoid reactions have been rarely reported after administration of non-steroid anti-inflammatory

pain reliever in such patients)

(4) Patient with asthma exposed to risk of increase in asthma severity, hives, or acute rhinitis due to aspirin or other drugs inhibiting other prostaglandin compounds

(5) Treatment of pain before and after CABG

(6) Patient with severe cardiac failure

(7) Patient with severe renal disorder

(8) Patient with severe liver disorder

(9) Patient with medical history of gastrointestinal tract hemorrhage or perforation due to non-steroid anti-inflammatory pain reliever

(10) Pregnant woman in late stage of pregnancy

(11) Breastfeeding woman

(12) Patient with inflammatory intestinal disease such as Chron's disease or ulcerative colitis

(13) Patient with hemorrhage or blood coagulation disorder

3. Administer with caution to following patients

(1) Patient with renal disorder or such medical history

(2) Patient with fluid retention or cardiac failure

(3) Patient administered diuretic or ACE inhibitor

(4) Patient in recovery period after surgical treatment

(5) Elderly

(6) Patient with bronchial asthma or such medical history(Bronchospasm has been reported as result of administering non-steroid anti-inflammatory pain reliever)

(7) Patient with inherited porphyrin metabolism disorder

(8) Patient with high blood pressure

(9) Patient with liver disorder or such medical history

(10) Patient with medical history of digestive tract ulcer due to long term administration of non-steroid anti-inflammatory pain reliever who requires long term administration of this drug and being treated for digestive ulcer with misoprostol and such(There are digestive ulcers resistant to misoprostol treatment so observe progress while administering this drug.)

(11) Women that may be in early or intermediate pregnancy stage, women that may be pregnant, or women that are planning for pregnancy

(12) Patient administered anticoagulant

4. Adverse Reactions

Most frequently reported adverse reactions were adverse gastrointestinal reactions, dizziness, and abnormal liver enzyme levels. Rare instances of adverse skin reactions including itch and hives were reported.

(1) Blood and lymphatic system : Rare instances of anemia and very rare instances of white blood cell count decrease, platelet count decrease, neutropenia, hemolytic anemia, aplastic anemia, agranulocytosis

(2) Hypersensitivity : Rare instances of shocks and anaphylactic reaction

(3) Metabolism : Very rare instances of high blood calcium level

(4) Peripheral nervous system : Frequent instances of dizziness and very rare instances of depression, strange dreams, insomnia

(5) Sensory system : Rare instances of visual impairment and optic neuritis and very rare instances of dysaesthesia, tremor, drowsiness, headache, dysgeusia, neck paralysis, fever, disorientation, derangement, hallucination, tinnitus, weariness Circulatory system : Very rare instances of palpitation and face flush. High blood pressure and cardiac insufficiency has been reported related to non-steroid anti-inflammatory pain reliever. Clinical trial and epidemiological data on non-steroid anti-inflammatory pain reliever(Especially high dosage and long term use) show that it may increase the risk of thrombosis(Ex. Arteriosclerosis or cerebral stroke).

(6) Respiratory system : Rare instances of dyspnoea and very rare instances of bronchospasm, stridor

(7) Digestive system : Most frequently, adverse gastrointestinal reactions are observed. Digestive ulcer, perforation, or gastrointestinal hemorrhage may occur which may be severe at times and it is more severe among elderlies. Frequent instances of indigestion, stomachache, nausea, diarrhea and occasional instances of abdominal inflation, gastritis, constipation, vomit, oral ulcer and very rare instances of stomatitis, hemoptysis, pancreatitis, aggravation of colitis, Chron's disease

(8) Liver : Very rare instances of hepatitis, jaundice, AST/ALT/ALP elevation

(9) Skin : Occasional instances of itch, hives, rubefaction, dermatitis, rashes and rare instances of facial edema and very rare instances of bullosa including peliosis, dermatitis bullosa, mucocutanealocular syndrome(Stevens-Johnson Syndrome), and toxic epidermal necrolysis (Riehl Syndrome), light hypersensitivity, hair loss

(10) Kidney : Renal failure, nephrotic syndrome

(11) Local reaction : Edema, fatigue, leg spasm

(12) Other : Frequent instances of liver enzyme level elevation, occasional instances of blood urea level or creatinine increase, weight increase, increase of nocturnal enuresis, autoimmune disease such as aseptic meningitis(SLE and MCTD)

(13) Following adverse events are added according to analysis and evaluation of domestic side effect report data.

- Skin: Angioedema
- Other: Peripheral edema, edema around the eye

5. General Caution

(1) Consider the potential risk and benefit of this drug and other substitute treatment method before administering this drug. This drug should be administered at minimum effective dosage over the shortest period possible consistent with purpose of treatment for each patient.

(2) Adverse gastrointestinal reaction: Extreme caution in prescription of non-steroid anti-inflammatory pain relievers including this drug should be exercised for patient with ulcerative disease or history of gastrointestinal hemorrhage. Patient with digestive ulcer disease and/or history of gastrointestinal hemorrhage is exposed to risk of gastrointestinal hemorrhage more than 10 times upon administration of non-steroid anti-inflammatory pain reliever compared to a patient without such risk factor. Other risk factors which increase the risk of gastrointestinal hemorrhage are co-administration with oral corticosteroid or anticoagulant, long term use of non-steroid anti-inflammatory pain reliever with alcohol consumption, old age, and weak physical state. Most of voluntary reports regarding fatal gastrointestinal reactions come from elderlies and physically weak people so administration of this drug for such patients should be cautious.

(3) High blood pressure: Non-steroid anti-inflammatory pain relievers including this drug may cause high blood pressure or aggravate existing high blood pressure and such may result in increase in occurrence of cardiovascular failures. Patient administered thiazide diuretic or loop diuretic whom are medicated with non-steroid anti-inflammatory pain reliever may experience reduced reaction from such treatment. Non-steroid anti-inflammatory pain relievers including this drug must be administered with caution for high blood pressure patients. Blood pressure must be closely monitored during

early stage of administration and throughout the administration period for this drug.

(4) Congestive heart failure and edema: Fluid retention and edema were observed in some patients medicated with this drug and other non-steroid anti-inflammatory pain reliever. This drug should be administered with caution for patients with fluid retention or cardiac insufficiency.

(5) Long term administration of non-steroid anti-inflammatory pain reliever may cause renal papillary necrosis or other renal damage. Also, special caution is required for cardiac insufficiency patients, renal function disorder patients, liver function disorder patients, patients administered with diuretic or ACE inhibitor, and elderlies since the role of prostaglandin is critical for maintaining RBF. Most patients should recover to state before treatment once administration is suspended.

(6) Progressing renal disease: There are no controlled clinical trials regarding usage of this drug on patients with progressed renal disease. Therefore, administration of this drug is not recommended for patients with progressed renal disease. Renal function of the patient should be monitored closely if administration of this drug is necessary.

(7) Administration of non-steroid anti-inflammatory pain relievers including this drug may result in elevated liver function levels. Such abnormal test results may get worse or remain the same or even be temporary as the treatment continues. Also, severe adverse reactions related to the liver including jaundice, fatal fulminant hepatic failure, hepatonecrosis, and hepatic insufficiency(Some can be fatal) were rarely reported for administration of non-steroid anti-inflammatory pain relievers including this drug.

(8) Patient with symptoms and/or signs of abnormal liver function or patient with abnormal liver function test results should be observed carefully throughout the administration period for aggravation of liver function abnormality and administration of this drug should be stopped when clinical symptoms or systemic signs(Ex: Acidophilia, hives) related to liver disease occur. Administration of non-steroid anti-inflammatory pain relievers including this drug may cause anemia so patients must be tested for hemoglobin count or HCT count when they exhibit symptoms or signs of anemia due to long term administration of this drug.

Non-steroid anti-inflammatory pain relievers suppress platelet aggregation and it has been confirmed to extend hemorrhage time in some patients.

Unlike aspirin, impact of this drug on platelet function is comparatively small, short in duration, and reversible. Patients that may be negatively affected due to change in platelet function from co-administration of anticoagulant or with coagulation related disease should not be administered.

(9) Consider the following when using for chronic disease.

① Patients administered non-steroid anti-inflammatory pain reliever long term must undergo regular CBC and physicochemical tests. Stop the administration of this drug should clinical symptoms related to liver or renal diseases or systemic signs(Ex: Acidophilia, hives) is observed or if liver function test or renal function test results continue to be abnormal or worsen.

② Consider treatment methods aside from drug treatment.

(10) Consider the following when using for acute disease.

① Medicate considering acute pain and fever severity.

② Avoid long term administration of same drug by principle.

③ Perform causal treatment if applicable.

(11) Anaphylactoid reaction: Anaphylactoid reaction may occur in patients without previous exposure to the substance like other non-steroid anti-inflammatory pain reliever. Such complex symptom may or may not accompany nasal polyp after administration of aspirin or other non-steroid anti-inflammatory pain reliever or occur in asthma patients exhibiting symptoms of potentially fatal bronchospasm. First aid should be administered when such anaphylactoid reaction is observed.

(12) Skin reaction: This drug may cause serious adverse skin reaction such as deciduous dermatitis, Stevens-Johnson syndrome, and toxic epidermal necrolysis and they may be fatal. Such serious adverse reactions may occur without warning symptoms. Most of the time, such reactions occur within 1 month of initial administration. Patients should be aware of serious skin symptoms and signs and stop administration of this drug when skin rashes or other initial symptoms and signs of hypersensitivity occur.

(13) Some asthma patients may be sensitive to aspirin. Use of aspirin for aspirin sensitive asthma patient may be result in severe bronchospasm which may be fatal. Cross-reaction including bronchospasm between aspirin and other non-steroid anti-inflammatory pain relievers in such aspirin sensitive patients has been reported. Therefore, this drug should

not be administered to such aspirin sensitive patients and be administered with caution for asthma patients.

(14) This drug cannot substitute corticoid agents or be used as a drug for treating corticoid deficiency. Sudden stoppage in corticosteroid administration may cause aggravation of corticosteroid-responsive disease. Administration of this drug for a patient administered with corticosteroid long term requires gradual reduction of dosage.

(15) Other symptoms and signs of inflammation may become unapparent due to the nature of this drug's pharmacological properties thus delaying the diagnosis of infectious complications through pain and noninfectious conditions.

(16) Patients experiencing other central nervous disorders(Drowsiness, fatigue, visual impairment) should avoid driving or handling machinery as well as environment requiring special attention.

(17) This drug may decrease fertility of women so women planning for pregnancy are recommended to avoid administration. Temporary infertility has been reported in women administered non-steroid anti-inflammatory pain reliever long term. Women having difficulty with pregnancy or undergoing infertility treatment are recommended to stop administration of this drug.

(18) Keep in mind that treatment by anti-inflammatory pain reliever is not a causal treatment but a symptomatic treatment.

6. Interaction

(1) Patient taking other drugs especially digoxin, anticoagulant, oral diabetic drug, or diuretic should consult a physician.

(2) ACE inhibitor/Angiotensin Ⅱ receptor antagonist : There are reports of reduced antihypertensive effect for ACE inhibitor or angiotensin II receptor antagonist due to non-steroid anti-inflammatory pain reliever so such interaction should be kept in mind for co-administration with ACE inhibitor or angiotensin II receptor antagonist.

(3) Aspirin : There is no consistent evidence that co-administration with aspirin reduces the risk of severe cardiovascular thrombus related to use of non-steroid anti-inflammatory pain reliever. Co-administration of this drug and aspirin may increase the risk of severe adverse gastrointestinal reaction like other non-steroid anti-inflammatory pain reliever so co-administration

of these two drugs is generally not recommended.

(4) Diuretic

① It was confirmed that the natriuresis effect of furosemide and thiazide diuretics may decrease in some patients due to prostaglandin synthesis inhibitor effect from this drug on the kidney after clinical trial and follow-up study after commercialization. Co-administration of such substances and non-steroid anti-inflammatory pain reliever requires close observation for signs of renal failure.

② Co-administration with potassium sparing diuretic may increase blood serum potassium level thus requiring monitoring of blood serum potassium level. It is yet unknown whether co-administration with bendrofluazide impacts blood pressure control but its interaction with other diuretics cannot be outruled.

(5) Lithium : Non-steroid anti-inflammatory pain reliever increases blood serum lithium level and decrease of renal clearance rate of lithium due to inhibition of prostaglandin synthesis by the kidney. Therefore, signs for lithium toxicity should be monitored when co-administering lithium and non-steroid anti-inflammatory pain reliever.

(6) Methotrexate: Co-administration with non-steroid anti-inflammatory pain reliever may delay excretion of methotrexate from renal tubule which may increase blood chemistry toxicity of methotrexate to a fatal level so co-administration of high dosage used for anticancer therapy(Above 15mg/week) is prohibited and co-administration with low dosage of methotrexate should be administered carefully.

(7) Coumarin anticoagulant(Warfarin, etc.) : Warfarin and non-steroid anti-inflammatory pain reliever may have elevated effect regarding gastrointestinal hemorrhage so patient using both substances may be more exposed to risk of severe gastrointestinal hemorrhage compared to a patient administered with just a single substance.

(8) Co-administration with other non-steroid anti-inflammatory pain reliever(Includes selective COX-2 inhibitor) is prohibited since it could increase the risk of adverse event.

(9) This drug is metabolized through CYP2C9 so it may have pharmacokinetic interaction with phenytoin, cimetidine, miconazole, sulfaphenazole, amiodarone, tolbutamide, and phenylbutazone.

(10) Cardiac glycoside: Non-steroid anti-inflammatory pain reliever reduces

glomerular filtration rate and increases blood glycoside level which may aggravate cardiac insufficiency.

(11) Mifepristone : Non-steroid anti-inflammatory pain reliever should not be administered for 8~12 days after administration of mifepristone as it reduces the effect of mifepristone.

(12) Quinolones : Take caution as there is animal test data reporting that co-administration with non-steroid anti-inflammatory pain reliever may increase the risk of spasm.

(13) Antiplatelet drug and SSRI : Co-administration with these drugs may increase the risk of gastrointestinal hemorrhage.

(14) Cyclosporine and tacrolimus : Co-administration with non-steroid anti-inflammatory pain reliever may increase the risk of toxic nephrosis.

(15) Zidovudine : Co-administration with non-steroid anti-inflammatory pain reliever may increase blood chemistry toxicity. There is evidence that co-administration of zidovudine and ibuprofen for HIV(+) hemophiliac patients increased the risk of hemarthrosis and hematoma.

(16) Diabetic drug: There were clinical results that diclofenac does not have impact on clinical effect of oral hypoglycemic agent when co-administered with oral hypoglycemic agent but effects of low blood sugar and high blood sugar were rarely reported during treatment with this drug so dosage for hypoglycemic drug should be adjusted appropriately.

(17) Corticosteroid: Co-administration with this drug may increase the risk of gastrointestinal ulcer or hemorrhage.

7. Administration for pregnant and breastfeeding women

(1) There is no clinical data on administration of this drug for pregnant women. Administration of this drug may prematurely shut down the arterial tube of the embryo towards late stage of pregnancy like other non-steroid anti-inflammatory pain reliever so this drug should be administered at minimum dosage with caution for women planning for pregnancy or in early/intermediate stage of pregnancy and administration of this drug should be restricted for pregnant women in late stage of pregnancy(Last trimester).

(2) Safety for pregnant women has not been sufficiently established with current data. Test on rats for this drug revealed increased frequency of dystocia like other drugs that inhibit synthesis of prostaglandin as well as

delay in childbirth and decrease in survival of newborn.

(3) Safety for breastfeeding women has not been sufficiently established with current data and it is not yet known whether this drug is secreted to breastmilk of human. However, many drugs not only transfer to breastmilk but there are also risks of severe adverse events for babies so breastfeeding or administration of this drug should be stopped considering the importance of drug administration for breastfeeding women.

8. Administration for children

Dosage or indicant is not designated for children(Safety and effectiveness of this drug has not been established for children and youth).

9. Administration for elderlies

Adverse reactions due to non-steroid anti-inflammatory pain relievers increase in terms of frequency especially for gastrointestinal tract hemorrhage and perforation. Therefore, administration of this drug for elderlies should be performed with caution like other non-steroid anti-inflammatory pain relievers.

10. Remedy for overdose

Typical symptoms of overdose are not yet known but treatment should be performed according to observed symptoms for overdose by mistake and the patient must prepare for gastrointestinal tract irritation, low blood pressure, hypoventilation, spasm, etc.

11. Storage and handling precaution

(1) Keep out of children's reach

(2) Changing container may cause accidents and it is not recommended for quality maintenance

11.1.3 Adverse Reactions and Use Precaution for Tylenol ER Tablet

1. Warning

1) A person regularly drinking alcohol in excess of three glasses per day who is medicating with this drug and other fever and pain reliever must consult with a physician or a pharmacist. Medication of this drug for such person may cause gastrointestinal hemorrhage.

2) Severe skin reactions such as AGEP, Stevens-Johnson Syndrome, and

TEN were reported very rarely amongst patients medicated with acetaminophen and such severe skin reactions may be fatal. Therefore, patients should be notified of symptoms for such severe skin reactions and patients should stop administration of this drug immediately upon skin rashes and other symptoms of hypersensitivity exhibited after administration of this drug.

2. Do not administer to following patients
1) Patient with hypersensitivity to this drug
2) Patient with peptic ulcer
3) Patient with severely abnormal blood levels
4) Patient with severe liver disorder
5) Patient with severe renal disorder
6) Patient with severe cardiac insufficiency
7) Patient with aspirin asthma(Asthma shock caused by non-steroid anti-inflammatory drug) or such medical history
8) Patient medicated with following substance : Barbital drugs, tricyclic antidepressants
9) Person under influence of alcohol
10) Children below age 12

3. Following activities are restricted during medication
1) Do not administer exceeding recommended dosage. Administration exceeding recommended dosage for this drug may result in liver damage.
2) Do not administer with other products including acetaminophen.
3) This drug is a slow release tablet so it should be swallowed without breaking, chewing, or dissolving.

4. Following patients should consult with physician, dentist, or pharmacist before taking this drug
1) Patient with liver disorder or such medical history
2) Patient with renal disorder or such medical history
3) Patient with history of peptic ulcer
4) Patient with abnormal blood levels or such medical history
5) Patient with tendency to hemorrhage(May exhibit platelet function disorder)

6) Patient with heart disorder
7) Patient with medical history of hypersensitivity
8) Patient with bronchial asthma
9) Elderly(Senior citizen)
10) Pregnant/Breastfeeding woman
11) Patient medicated with warfarin long term
12) Patient medicated with following substances : Lithium, thiazide diuretic

5. Stop taking this drug immediately and consult with physician, dentist, or pharmacist under following circumstances. Bring this attached document for consultation if possible.
1) Shock: Shock, anaphylactoid symptoms(Symptoms similar to hypersensitivity : Dyspnoea, whole body turning red, blood vessel swelling, rashes, etc.), asthmatic attack
2) Blood: Platelet count decrease, granulocyte count decrease, hemolytic anemia, methemoglobinemia, platelet function decrease(Increase of hemorrhage time), cyanosis
3) Hypersensitivity: Hypersensitivity symptoms(Facial swelling, dyspnoea, sweating, low blood pressure, shock)
4) Digestive system: Nausea, vomit, appetite loss, adverse gastrointestinal reactions such as gastrointestinal hemorrhage, digestive ulcer, perforation when administered long term
5) Skin: Hives, allergic reactions, mucocutanealocular syndrome(Stevens-Johnson Syndrome), toxic epidermal necrolysis(Riehl Syndrome)
6) Other: Chronic liver necrolysis, acute pancreatitis, chronic hepatitis, and toxic nephrosis for long term administration
7) Overdose: Necrosis of liver, kidney, and heart
8) Adverse events reported in clinical trial were listed in the descending order of frequency by body part in Chart 1 below. Cause-and-effect relationship with this drug was not taken into consideration. Frequency is as following: Very common($\geq$ 1/10), common ($\geq$ 1/100, $<$ 1/10), sometimes($\geq$ 1/1,000, $<$ 1/100), rarely ($\geq$ 1/10,000, $<$ 1/1,000).

**Immune system disorder**<br>Very rare : Anaphylactic reaction, hypersensitivity<br><br>**Skin and subcutaneous tissue disorder**<br>Very rare : Hives, Pruritogenic(Itch) rashes, rashes

Chart. Adverse Reactions Discovered After Commercialization According to Frequency Based on Voluntary Report Rate

9) Add the following adverse reactions based on analysis and assessment of domestic report data of adverse reactions.
   - Liver and gall bladder: AST elevation, ALT elevation
   - Skin: Fixed eruption

6. Other precautions when taking this drug

1) General caution
   (1) Receive sufficient consultation to predict symptoms of hypersensitivity.
   (2) Keep in mind that treatment by anti-inflammatory pain reliever is not a causal treatment but a symptomatic treatment(Treatment by symptom)
   (3) Consider the following when using for chronic disease.
   A) Take regular clinical examinations(Urine test, blood test, liver function test) for long term administration and take appropriate action for any abnormality such as dosage reduction and suspension of administration.
   B) Consider treatment methods aside from drug treatment.
   (4) Consider the following when using for acute disease.
   A) Medicate considering acute pain and fever severity.
   B) Avoid long term administration of same drug by principle.
   C) Perform causal treatment if applicable.
   (5) Children and elderlies should be administered minimum required dosage and monitored with caution for adverse reactions. They may exhibit excessive body temperature drop, collapse, limb ischemia, and other symptoms so monitor the status of children and elderlies or wasting disease patients accompanied by high fever with caution.
   (6) Avoid co-administration with other anti-inflammatory pain relievers.
   (7) This drug shall not be administered for more than 3 days for fever and more than 10 days(Adult) for pain without instruction from a physician or a pharmacist. Consult with a physician or a pharmacist if the pain or fever symptoms continue or become aggravated.

(8) Infection may not be visible on the exterior when taking this drug so patients with infection complications should be administered with appropriate antimicrobial according to prescription.

2) Remedy for overdose

Overdose of this drug requires immediate medical treatment even without clear signs or symptoms. Protect the liver through intravenous injection of N-acetylcysteine or oral administration of methionyl within 10~12 hours.

7. Caution for Storage

1) Store at room temperature(1~30℃) in sealed container
2) Keep out of children's reach
3) Displacing the drug from original container may cause accident or deterioration in quality so store in the sealed original container.

11.2 ACR Diagnosis Criteria

American College of Rheumatology classification criteria for osteoarthritis of the knee

<Traditional Format>

When knee pain and radiographic osteophytes are observed or when a patient meets one of the following conditions;

·Age > 50 years
·Morning stiffness ≤ 30 minutes in duration
·Crepitus on motion

<Classification Tree>

When knee pain and radiographic osteophytes are observed or when a patient meets all three of the following conditions;

·Age ≥ 40 years
·Morning stiffness ≤ 30 minutes in duration
·Crepitus on motion

11.3 Kellgren & Lawrence I-IV X-ray Grading

Stage		Criteria
Stage I	:	Slight rarefaction of the trabecular structure in the head of the tibia. Sighs of an axis anomaly. Joint space still appears normal. Pointing of the intercondylar tubercles. Possible slight demineralization of the patella.
Stage II	:	Joint space asymmetrically narrowed. Axis anomaly (varus valgus). Osteophytes on the patella, the head of the tibia and intercondylar tubercles.
Stage III	:	Degeneration of the cancellous bone in the head of the tibia with formation of small cysts. Increasing alternation of the joint space width and osteophytes.
Stage IV	:	Joint space virtually absent, malposture, deformation of the femoral condyles with osteophytes of bizarre shapes, chondral debris cyst, instability of the joint.

11.4 K-WOMAC Scale

Pain

Please tell us how severe the pain caused by arthritis was over the past 48 hours.

	Question	None (0)	Slightly (1)	Average (2)	Severe (3)	Very Severe (4)
1	When walking on flat ground					
2	When walking up and down the stairs					
3	Disturbed by pain that interrupts sleep when sleeping at night					
4	When sitting (On a chair) or when lying down					
5	When standing upright					

Stiffness

Please tell us how severe the stiffness caused by arthritis was over the past 48 hours. Stiffness refers to instances when the joints are not moving easily when moving.

	Question	None (0)	Slightly (1)	Average (2)	Severe (3)	Very Severe (4)
1	How severe is the joint stiffness when you wake up in the morning?					
2	How severe is the stiffness you feel after sitting (on a chair), lying down, or resting in the afternoon?					

Daily Life Difficulties

Please tell us how severe the difficulty in daily life caused by arthritis was over the past 48 hours. Daily life capabilities refer to the patient's mobility and the ability to care for self.

	Question	None (0)	Slightly (1)	Average (2)	Severe (3)	Very Severe (4)
1	When going down the stairs					
2	When going up the stairs					
3	When sitting (On a chair) and standing up					
4	When standing					
5	When bending over towards the floor					
6	When walking on flat ground					
7	When boarding or getting off the passenger vehicle or the bus					
8	When going grocery shopping					
9	When putting on socks or stockings					
10	When rising from bed					
11	When taking off socks or stockings					
12	When going to bed					
13	When going in and out of the bathtub					
14	When sitting (On a chair)					
15	When sitting on or standing up from the toilet					
16	When performing physically stressful labor					
17	When performing light physical labor					

12. LIST OF ATTACHMENTS

Attachment 1. Description and Agreement for Subject

Attachment 2. Regulation on Victim Compensation

Attachment 3. Clinical Trial Institutions and Administrators
